# Profiling Environmental Variations in Condensed Tannins and Other Metabolites of Birdsfoot Trefoil (*Lotus corniculatus* L.) Genotypes

**DOI:** 10.3390/plants14172766

**Published:** 2025-09-04

**Authors:** Solihu Kayode Sakariyahu, Tim McDowell, Justin B. Renaud, Yousef Papadopoulos, Kathleen Glover, Rebecca Nelson Brown, Michael D. Peel, Heathcliffe Riday, Susanne E. Kohalmi, Abdelali Hannoufa

**Affiliations:** 1 London Research and Development Center, Agriculture and Agri-Food Canada, London, ON N5V 4T3, Canada; solihukayode.sakariyahu@agr.gc.ca (S.K.S.); tim.mcdowell@agr.gc.ca (T.M.); 2 Department of Biology, University of Western Ontario, London, ON N6A 3K7, Canada; skohalmi@uwo.ca; 3 Department of Biology, Ahmadu Bello University, Zaria 810107, Nigeria; 4 Kentville Research and Development Centre, Agriculture and Agri-Food Canada, Kentville, NS B4N 1J5, Canada; yousef.papadopoulos@agr.gc.ca (Y.P.); kathleen.glover@agr.gc.ca (K.G.); 5 Department of Plant Sciences and Entomology, University of Rhode Island, Kingston, RI 02881, USA; brownreb@uri.edu; 6 USDA-ARS Forage and Range Research Laboratory, Utah State University, Logan, UT 84321, USA; mike.peel@ars.usda.gov; 7 USDA-ARS US Dairy Forage Research Center, Madison, WI 53706, USA; heathcliffe.riday@usda.gov

**Keywords:** birdsfoot trefoil, chromatographic analyses, forage quality, geographical location, kaempferol, mass spectrometry, metabolites, phytochemistry, phytochemical profile, procyanidins, prodelphinidins, tannins

## Abstract

*Lotus corniculatus* L., also known as birdsfoot trefoil (BFT), is a perennial, non-bloating, temperate forage legume widely grown due to its accumulation of high levels of condensed tannins (CTs) in foliage. However, variations in the CT levels and other plant metabolites in BFT genotypes in response to environmental and yearly factors under field conditions remain largely unexplored. Here, we combine conventional CT quantification and metabolome profiling with high-resolution liquid chromatography–mass spectrometry (LC-MS) to understand how environmental factors impact CT and other metabolite profiles. Eight BFT genotypes grown in Kentville, Canada, and Rhode Island and Utah in the United States were investigated, revealing significant genotypic variations in soluble CT contents. The global metabolome profiles of the eight BFT genotypes clustered predominantly based on geographical location. These results demonstrate that geographical location strongly influences CT accumulation and metabolome composition, offering potential for selecting genotypes adapted to specific environments. Our findings provide an opportunity for targeted breeding strategies to optimize CT levels, improve forage quality, and enhance stress resilience in birdsfoot trefoil.

## 1. Introduction

Grassland livestock production relies heavily on high-quality forage to maintain productivity and animal health [1]. Of the pasture species, forage legumes are the most important in North American grassland, as they provide high crude protein, palatability, and digestibility, in addition to a rich source of specialized metabolites [2,3]. Forage legumes can also fix atmospheric nitrogen through symbiotic relationships with rhizobia bacteria, reducing the need for synthetic nitrogen fertilizers. The relatively deep root systems of these plants allow them to access moisture from deeper soil layers, resulting in moderate drought tolerance. These traits help improve biodiversity, soil health, and the potential for carbon sequestration in the pasture environment [4]. Despite these beneficial characteristics, consumption of some leguminous forages, such as alfalfa (*Medicago sativa* L.) and clover (*Trifolium* spp.), which are rich in soluble proteins, can lead to a potentially dangerous condition known as pasture bloat in ruminants. Bloating is characterized by the accumulation of gases in the rumen and occurs when the normal processes of eructation (belching) are disrupted, leading to death in severe cases and non-lethal symptoms such as frequent urination, respiratory distress, and large production of methane gas [5,6]. Although methane gas is relatively unstable in the atmosphere (half-life < 12 years), it is nevertheless one of the largest components of the livestock industry’s contribution to greenhouse gas emissions [7,8]. Incorporating condensed tannins (CTs) containing forage legumes, such as sainfoin (*Onobrychis viciifolia* Scop.) and birdsfoot trefoil (BFT; *Lotus corniculatus* L.), into forage pasture helps reduce bloating in ruminants [9]. The anti-bloating attributes of sainfoin and birdsfoot trefoil are due to their possession of substantial amounts of CTs, which are largely lacking in alfalfa and clover [9,10].

BFT is a widely cultivated forage legume containing CTs at levels ranging from 30 to 40 g/kg dry matter [11,12,13]. BFT has several agronomic benefits, including the ability to grow in acidic and poorly drained soils [14]. In pastures composed of cool-season grasses, BFT forage will continue to grow and produce during the summer slump, whereas the cool-season grasses go dormant [15]. It was also shown to improve the absorption of amino acids and enhance reproductive rates and milk secretion in sheep [16,17,18]. For instance, a study by Hymes-Fecht et al. [19] demonstrated that BFT lowers ruminal concentrations of ammonia and free amino acids, suggesting it could reduce the formation of stable protein foams and the proteolysis of dietary protein [9,20]. Moreover, in a previous field study, cattle grazing on BFT pastures had a lower occurrence of bloat than animals grazed on alfalfa alone [21]. Another potential benefit of BFT as a forage is the apparent preventative and therapeutic effects against pathogenic nematodes. BFT forage has been shown to influence *Haemonchus contortus* infection by reducing the total worm burden and fecal egg count in sheep [22,23]. A study looking at the activity of three tannin-containing forage species found that many showed antiparasitic activity against *H. contortus*; yet, no significant correlation was observed between the CT levels of the forage and anti-nematode activity [24]. Nevertheless, BFT adoption as forage has been ascribed to its anti-bloating and antiparasitic properties that come from its CT content and composition.

Tannins are polyphenolic specialized metabolites found across the plant kingdom. They encompass two main groups: CTs (also known as proanthocyanidins or PAs) and hydrolyzable tannins [25,26]. CTs are formed through the polymerization of flavan-3-ols units such as catechin, epicatechin, gallocatechin, and epigallocatechin. These units link together to form oligomers (2–10 units) or polymers (>10 units), with polymeric forms, such as procyanidins (oligomers of catechin and epicatechin) and prodelphinidins (oligomers of gallocatechin and epigallocatechin) [27] often being more abundant in plant tissues. In contrast, hydrolyzable tannins are esters of gallic acids or their derivatives with a sugar core, typically glucose, and can yield ellagic acid upon hydrolysis [26]. The biosynthesis of CTs occurs via the phenylpropanoid and flavonoid pathways, with key enzymes like leucoanthocyanidin reductase (LAR) and anthocyanidin reductase (ANR) catalyzing the formation of flavan-3-ol monomers that are subsequently polymerized. CTs were initially thought to only play a role in plant defense against herbivory by making the plant tissue less digestible and palatable for insects. However, recent research has revealed that CTs have diverse biological roles beyond herbivore defense, including protection against pathogens, allelopathic effects on neighboring plants, and potential human health benefits due to their antioxidant and other bioactivities [10,28]. Moderate levels of CTs have been shown to improve nutrient utilization in ruminants, including increasing the absorption of essential amino acids [9,29]. CTs can also directly reduce the risk of ruminant bloating by forming insoluble complexes with proteins, increasing rumen protein by-pass and digestion of forage [30,31]. The CT–protein complexes are then broken down under more acidic conditions in the abomasum, aiding plant-to-animal protein utilization by ruminants [18,32,33]. CTs also modulate the rumen microbiome, with certain CTs showing the ability to decrease the number of methanogens, thereby reducing methane emissions [34]. Higher levels of CTs, however, can have deleterious effects on ruminants, including reduced palatability, decreased feed intake, and lower nutrient utilization [9,35].

To effectively maximize the benefits of CT-containing forage to cattle, it is essential to understand the various factors that influence the profiles of their CTs and other metabolites that contribute to their bioactivities. Studies have shown that the CT composition, including the ratio of prodelphinidins (PDs) to procyanidins (PCs) and the degree of polymerization, can significantly affect their bioactivities and consumption by animals [36]. Furthermore, several factors have been reported to influence the composition of CTs in plants, including environmental conditions, seasonal changes, and the number of forage harvests [37,38,39,40]. For instance, it has been shown that the light intensity, temperature, soil nutrients, and water availability significantly affect the production of CTs in BFT [41,42,43,44,45]. A previous study examining several varieties of BFT grown at four locations in the United States found that both location and genotype significantly affected the CT contents [46]. There is high variability in the CT contents and compositions within different genotypes of CT-containing forage species such as *Lotus* spp. and sainfoin [12,39,40,47,48,49,50]. Various research studies have exploited the CT variability through intra- and inter-specific breeding efforts [51,52,53,54]. While there have been extensive efforts to understand the structural diversity, composition, and the effects of some environmental cues on CT accumulation, how genotype, geographical location, yearly variations, and their interactions influence the composition of CTs as well as other metabolites of BFT under field conditions remains largely understudied. Therefore, research is needed to elucidate these influences and optimize the use of CT-containing forage in diverse pastures and North American cool-season pasture settings.

The goal of the current study was to investigate the environmental and genotype-specific variation in accumulation of CTs and other metabolites of BFT under field conditions. We combined the conventional CT analysis method with high-resolution liquid chromatography–mass spectrometry (LC-MS) to measure the CT levels, composition, and other metabolites in eight BFT genotypes grown at different locations and in different years. We gained insight into how different geographical growth environments affect the levels of CTs and other metabolites that may play a role in the antiparasitic activities of birdsfoot trefoil.

## 2. Results and Discussion

### 2.1. Geographical Growth Location Influence on Condensed Tannin Levels in BFT Genotypes

**Figure 1:**
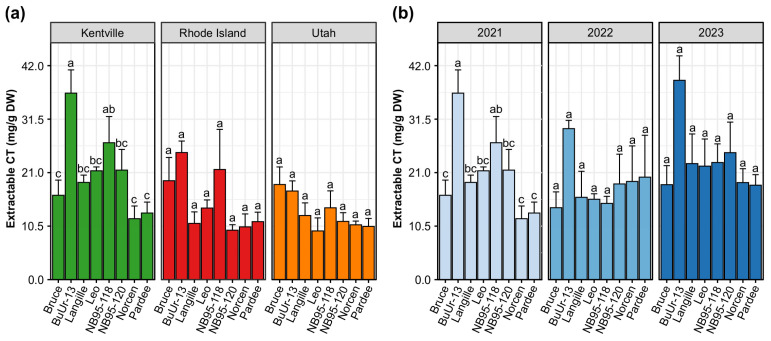
The condensed tannin contents in the eight birdsfoot trefoil genotypes across (**a**) three growth locations (Kentville, Rhode Island, and Utah) in 2021 and (**b**) three growth years in Kentville. Extractable condensed tannins were quantified by the butanol–HCl method in mg/g dried weight of BFT samples. Data are shown as the mean ± standard error of the mean (for *n* = 3–4 biologically independent replicates); the different letters above the bars represent statistical differences determined by analysis of variance on genotypes within each location and year (ANOVA; columns followed by a different letter are different, *p* < 0.05) using R 4.4.0.

Analysis of the extractable CTs across three locations in 2021 using the butanol–HCl colorimetric method revealed that levels ranged from 11.96 to 36.6 mg/g dry weight (DW) in Kentville compared to 9.70–24.95 mg/g DW in Rhode Island and 9.56–18.67 mg/g DW in Utah (Figure 1a). Across all locations, BuUr-13 exhibited the highest mean extractable condensed tannin content, particularly in Kentville, while Norcen, NB95-120, and Leo showed the lowest soluble CT values in Kentville, Rhode Island, and Utah, respectively. The ANOVA results for extractable condensed tannins of eight BFT genotypes from three locations in 2021 are presented in Table 1. The concentrations of extractable condensed tannins in the BFT forage varied significantly across genotypes (*p* < 0.001) and geographical locations (*p* < 0.001). No significant interaction effect was observed between the genotypes and geographical locations (Table 1). These results demonstrate that genotype and location have a significant influence on soluble CT concentrations, while their interaction does not, suggesting that genotypic differences in CT production are largely consistent across environments.

Further analysis of the year-to-year CT variation in Kentville revealed that year had a marginal effect on extractable CT levels (*p* = 0.057), while genotype remained a highly significant factor (*p* < 0.001), and no significant year × genotype interaction was observed (*p* = 0.910) (Table 1). Similarly, the genotype BuUr-13 had the highest extractable CT content across all the growth years in Kentville (Figure 1b), while there were no significant genotype effects on extractable CTs in Rhode Island or Utah (Figure S1a,b). Genotype had a more pronounced effect on extractable CT than growth year in Kentville compared to the other locations (Figure 1b and Figure S1d). The lack of genotypic and yearly differences in the CT levels in Utah may be attributed to the inherently lower and less variable accumulation of CTs in BFT genotypes grown at this location. The significant environmental influences on the CT content of BFT in this study are consistent with several previous studies on BFT, suggesting that differences in climatic factors, including soil nutrients, temperature, day length, and precipitation patterns, may be factors that affect the accumulation of CTs in their foliage. To a greater extent, there was a significant genotypic effect on the CT concentration of BFT, which indicates varying genetic capacities of different genotypes for synthesizing CTs in their foliage. The high CT levels observed in BuUr-13 across locations and years highlight its potential as a genetic resource for breeding programs aiming to enhance tannin-related traits, such as improved anti-bloating quality in forage crops [20,55]. Contrary to our initial expectation, year-to-year variation in Kentville had a minimal effect, indicating that environmental fluctuations within a single location may be less critical than broader geographic differences.

The two-way ANOVA revealed significant effects of location (*p* < 0.001), genotype (*p* < 0.001), and year (*p* < 0.001) on the insoluble CT concentrations, with no significant interaction effects (*p* > 0.05) (Tables S2a,b, S3a–d and S4). Across locations in 2021, NB95-120 and BuUr-13 exhibited the highest mean insoluble CT levels in Rhode Island and Utah, whereas Pardee and Bruce consistently had the lowest concentrations (Figure S2a). The yearly variation in Kentville showed a strong increase in insoluble CT contents from 2021 to 2023, with NB95-118 and NB95-120 reaching the highest levels of 141.50–143.64 mg/g in 2022–2023 (Figure S2b). While the soluble and insoluble CT levels were significantly influenced by both location and genotype, the overall soluble concentrations were markedly lower than the insoluble CT levels. The contrasting patterns observed between soluble and insoluble CTs suggest the differential allocation and partitioning of CTs in forage legumes. One possible explanation for the higher insoluble CTs in BFT could be that as tannins polymerize or bind to cellular components, such as cell walls or proteins, their solubility decreases, leading to higher insoluble CT accumulation [55,56].

### 2.2. Identification of BFT Metabolites Using Untargeted Metabolomic Analysis

**Figure 2:**
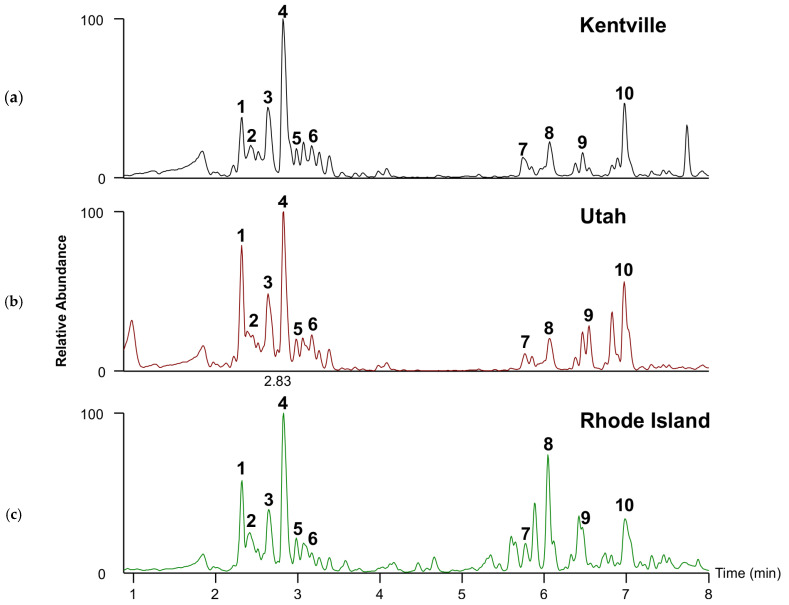
Base peak ion chromatograms of the metabolite profile in positive HESI mode of BFT across (**a**) Kentville, (**b**) Rhode Island, and (**c**) Utah in 2021. BuUr-13 is used as a representative genotype. Chromatograms represent the results of three independent analyses, all of which yielded similar outcomes. Compounds identified in the chromatogram are 1. tryptophan; 2. rutin; 3. kaempferol 3-rhamnoside-7-glucoside; 4. kaempferitrin; 5. coumaric acid; 6. kaempferol 7-O-rhamnoside; 7. soyasaponin I; 8. soyasaponin βg; 9. glucosyl-monolinolein; 10. diglucosyl-monolinolein.

**Figure 3:**
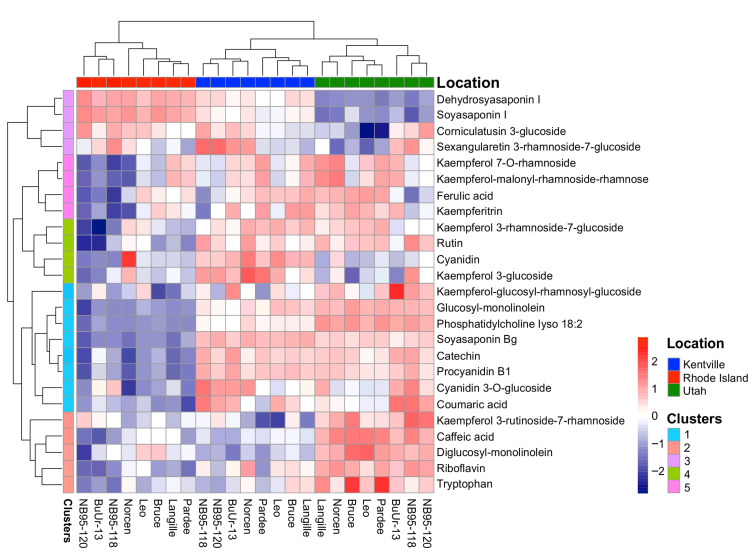
Heatmap showing the accumulation of metabolites in birdsfoot trefoil genotypes across Kentville, Rhode Island, and Utah in 2021. The scaled values in the heatmap are the average z-scores of the peak areas extracted from the LC-MS analysis of the BFT samples. The red in the heatmap indicates higher metabolite accumulation across locations, while blue indicates lower metabolite accumulation across locations.

In addition to the CT analysis, we also set out to identify additional metabolites in BFT. For this purpose, the metabolite profiles of the BFT samples were explored using a non-targeted analysis approach to assess the major compounds (peak height ≥ 1 × 10^7^ in all samples) in the extracts of BFT across the three growth locations in 2021. A total of 25 compounds throughout the studied samples were annotated at either a level 1 or 2 confidence (Table 2). LC-MS chromatograms showed that flavonol aglycones and flavonol glycosides (rt 2.00–3.5 min) and saponins (rt 5.80–7.5 min) were the most abundant specialized metabolites detected (Figure 2). While peak intensities in chromatographic analysis do not always directly correspond to absolute quantities, the predominant flavanol glycosides detected were kaempferol derivatives, specifically kaempferitrin (kaempferol 3,7-dirhamnoside) and kaempferol 3-rhamnoside-7-glucoside. These compounds, characterized by a kaempferol aglycone core, were the major constituents observed in the samples. The occurrence of a large accumulation of kaempferol and its glycosylated derivatives is not unexpected, as several reports have shown that kaempferol is one of the most diverse and abundant flavonoids in BFT extracts [43,57,58,59]. The predominant saponins identified were putatively characterized as soyasaponin I (C_48_H_78_O_18_) and soyasaponin βg (C_54_H_84_O_21_), based on their mass spectral data and molecular formulae (Table 2). These compounds, belonging to the group B soyasaponins, were the major triterpene glycosides detected in the samples. Metabolite profile analysis revealed that these compounds accumulated in a location-dependent manner (Figure 3). While it has been reported that levels of metabolite accumulation largely depend on environmental factors [60,61], these major metabolites were detected in all genotypes across sites, but their relative abundances varied distinctly across locations.

### 2.3. LC-MS Characterization of CT Compositions in BFT Genotypes

**Figure 4:**
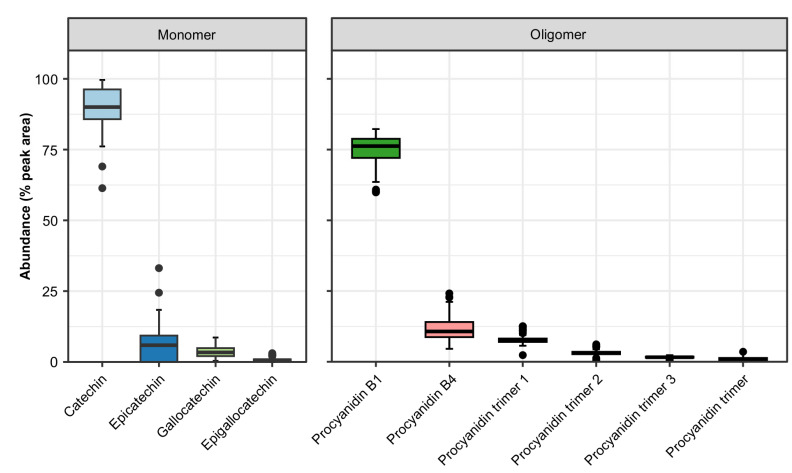
Box-and-whisker plot showing percent accumulations of CT monomers and oligomers in the forage of BFT. The peak areas of the EICs of catechin, epicatechin, gallocatechin, and epigallocatechin monomers were used to calculate the percentage accumulation of each monomer. Similarly, the peak areas of the EICs of procyanidin B1, procyanidin B4, procyanidin trimers, and prodelphinidin trimers were used to calculate the percent accumulation of each oligomer. The boxplot was prepared using % accumulation values from eight BFT genotypes from three locations in 2021. The whiskers on the plot indicate the standard errors.

Four monomeric CT building block compounds, catechin, epicatechin, gallocatechin, and epigallocatechin, were identified in the BFT extract in an approximate ratio of 120:8:5:1 (Table 3, Figure 4). Two dimeric and four trimeric proanthocyanidin metabolites were also identified, including procyanidin B1, procyanidin B4, three procyanidin trimers, and one prodelphinidin trimer (Table 3). Among the low-molecular-weight (MW) CT compounds, procyanidin B1 had the highest accumulation, followed by procyanidin B4, procyanidin trimer-1, and procyanidin trimer-2 in approximate proportions of 24:4:3:1, while procyanidin trimer-3 and the prodelphinidin trimer occurred only at trace levels (Table 3, Figure 4). The confidences of the metabolite identifications are listed in Table 3. The identification of compounds for which commercial standards were not available was aided by their respective MS/MS spectra (Table S5; Figures S7–S9). In the case of compounds based on either catechin or epicatechin building blocks, diagnostic product ions were indicative of the presence of catechin/epicatechin. Similarly, conjugated molecules based on kaempferol produced a diagnostic product ion of *m*/*z* 287.055 following the neutral losses of the glycosides.

Profiling of the 10 CT building block compounds revealed location-specific abundance with higher accumulation in Kentville compared to in Rhode Island and Utah (Figure 3). The composition of CTs found in this study is consistent with earlier reports in terms of the monomer composition and the presence of low-molecular-weight CTs in *Lotus corniculatus* [63,64]. However, the BFT CT composition appears less diverse than reports for *Lotus pendulum* and *Onobrychis viciifolia*, which showed significantly higher accumulations of prodelphinidins and higher-molecular-weight CTs [40,47]. These observed species–specific differences in CT diversity may be a major factor contributing to their varying degree of bioactivities against nematodes, protein-binding activities, and forage quality.

### 2.4. Influence of Geographical Location, Seasonal Factor, and Genotype on BFT Metabolomes

**Figure 5:**
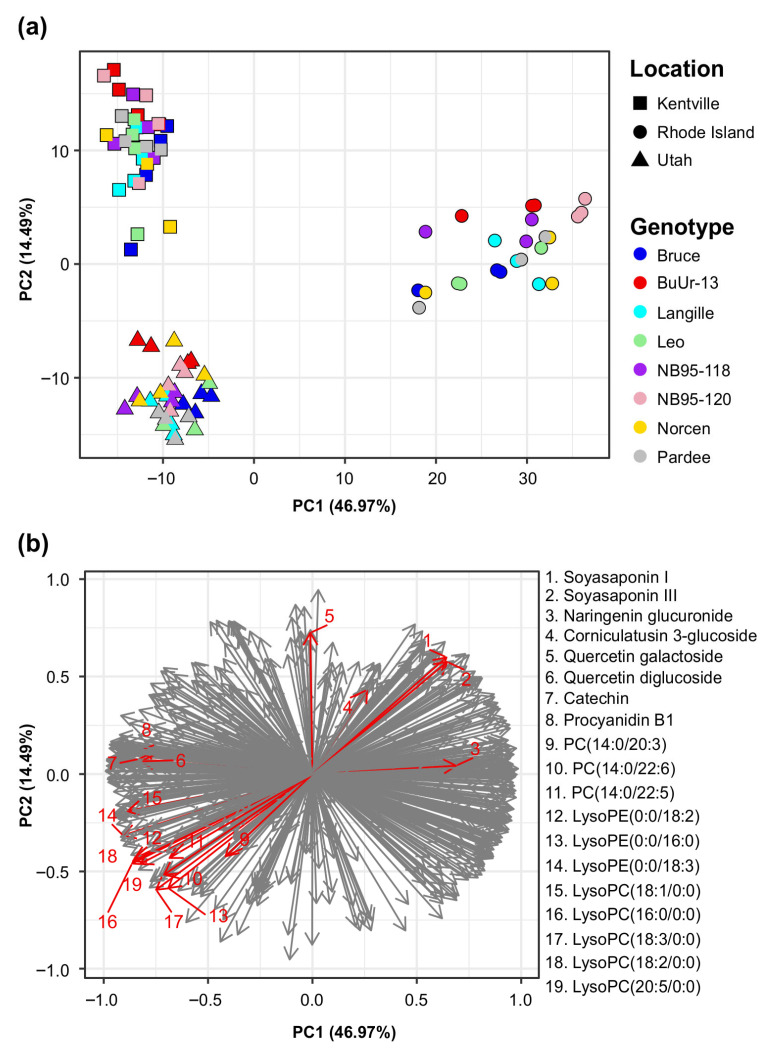
Principal component analysis (PCA) of 674 LC-MS positive-HESI-mode features from eight birdsfoot trefoil (BFT) genotypes across three environments. (**a**) PCA plot showing the location-specific clustering of the BFT genotypes. (**b**) Factor loading plot showing the features driving the location-specific clustering of the BFT samples. The red arrows indicate the most significant compounds driving the location-specific differences.

To determine whether genotype and geographical location influence the global metabolome of BFT, we performed a principal component analysis (PCA) on 674 molecular features found in positive HESI mode from extracts of the eight genotypes and three locations in 2021 (Figure 5a). Global clustering analysis of the BFT based on metabolite profiles revealed that growth locations had a stronger effect on the metabolomes of BFT than genotype. Notably, the PCA plot revealed that the BFT genotypes in this study were separated into three distinct clusters, corresponding to their geographical locations. The first principal component (PC1) of the PCA accounted for 46.97% of the variance and distinguished between genotypes grown in Rhode Island and those grown in Kentville and Utah. The second component (PC2) accounted for an additional 14.49% of the variance, separating genotypes grown in Kentville and Utah, as well as the genotype-specific differences within each location. Therefore, we were interested in identifying the metabolites that contribute to the observed grouping pattern. Further analysis of the loading plot revealed that several molecular features were responsible for separating Rhode Island BFT samples from those of Utah and Kentville along PC1 (Figure 5b). Metabolites such as catechin, procyanidin B1, and lysophospholipids, including PCs (phosphatidylcholines), lysoPEs (lysophosphatidylethanolamines), and lysoPCs (lysophosphatidylcholines), were mostly responsible for the separations along PC1 (Figure 5b, Table S5). In addition, glycosylated triterpenes and flavonoid compounds, such as soyasaponins, naringenin glucuronide, and corniculatusin 3-glucoside, were responsible for the distinct grouping of samples from Rhode Island. This grouping pattern prompted us to test for differentially accumulated metabolites (DAMs) using pairwise comparisons to detect metabolic features that were differentially accumulated when comparing the three geographical locations. A total of 461 out of 674 *m*/*z* features were found to be differentially accumulated in at least one pairwise comparison of locations. The highest numbers of DAMs were found between Kentville and Rhode Island, followed by Utah and Rhode Island, while the lowest numbers were between Kentville and Utah (Figure S5a–d), indicating that BFT genotypes grown in different locations had different metabolite profiles, consistent with the result from the principal component analysis (Figure 5a).

**Figure 6:**
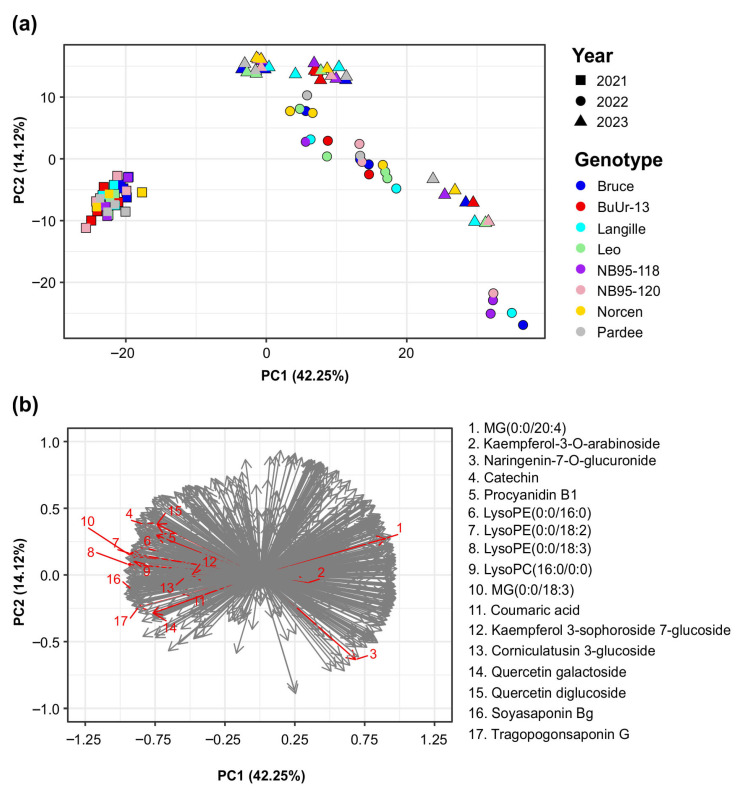
Principal component analysis (PCA) of 846 LC-MS positive-HESI-mode molecular features from eight birdsfoot trefoil genotypes across three growing years (2021, 2022, and 2023) in Kentville, Nova Scotia, Canada. (**a**) PCA plot of BFT samples showing year-specific clustering. (**b**) Factor loading plot showing the features driving the year-specific clustering of the BFT samples. The red arrows indicate the most significant compounds driving the location-specific differences.

Given that our CT analysis indicated that Kentville had the highest CT concentration variability within site among the three locations, the yearly variability in the metabolomic profile of BFT from 2021 to 2023 was explored. The PCA on 846 LC-MS molecular features of the extracts from eight genotypes and three growth years (Figure 6a) revealed that the samples clustered predominantly based on the year of sample collection, rather than by genotype. The first component of the PCA accounted for 42.25% of the variance and distinguished between genotypes grown in 2021, 2022, and 2023. The second component (PC2) accounted for an additional 14.12% of the variance. The loading plot analysis showed several metabolites with year-specific accumulation in Kentville, including CT-related compounds (such as catechin and procyanidin B1), lysophospholipids, flavonoid glycosides, and saponins (Figure 6b, Table S5). The highest number of DAMs was found between 2021 and 2022, with 502 DAMs, followed by 2021 and 2023, with 475 DAMs, while the lowest number was between 2022 and 2023, with 169 DAMs (Figure S6a–d), indicating that the BFT metabolome is influenced by the growth year in addition to the environment. Although the variations in the metabolome of BFT across years are less pronounced compared to the location effects, the distinct metabolite profiles reflect the changes in the environmental factors across different growth years (Tables S2 and S3).

Since climatic factors vary across different growing locations, BFT plants are expected to exhibit distinct CT contents and metabolome profiles at distinct geographic sites. The present study demonstrated the dependencies of the CT levels, compositions, and other metabolites in BFT on genotypic and geographical growth locations using conventional CT analysis and global metabolome profiling based on LC-MS. Our data indicate that genotype was the strongest predictor of CT content; however, the global metabolic variation in BFT may be linked to prevailing climatic factors at growing locations.

Examining extractable CT contents, the lowest levels were observed in BFT genotypes grown in Logan, Utah, which had the lowest total precipitation and/or artificial irrigation compared to Kentville and Rhode Island. Although soil moisture in Utah was supplemented with irrigation, the average soil water across the growing period may have been insufficient to support higher CT accumulation [65,66] compared to BFT grown in Kentville, which had the highest total precipitation among the three locations. Therefore, the levels of extractable CTs could be influenced by the total precipitation pattern and/or its distribution across the growing period. In contrast, our global metabolome clustering did not align with the differences in precipitation patterns. Instead, the BFT metabolomic profiles appear to be influenced by the mean temperatures across the growing locations, which reflect geographical site-specific climatic conditions, as Rhode Island experienced consistently warmer temperatures throughout the growing season compared to Kentville and Utah before sampling. This suggests that the metabolic reprogramming which led to the distinct metabolite profiles of BFT across the different growing locations may have resulted from temperature differences. Other factors, such as the soil nutrient availability, may contribute to variations in the CT contents and the distinct metabolic profiles of BFT, as nutrients are known to influence the production of plant specialized metabolites. Differences in soil fertility across locations may affect plant growth and metabolic pathways, interacting with genotypic effects to shape CT accumulation and metabolite profiles. Unfortunately, soil nutrient data were not collected in this study, limiting our ability to assess their impact. Therefore, future metabolomic experiments should prioritize investigating the role of soil nutrients, temperature, water limitation, and their combinatorial effects on the CT and metabolic profiles of BFT.

Taken together, genotypic, environmental, and year-specific variations in the CTs and metabolites of BFT reported in this study highlight the challenges to optimizing CT levels of BFT for improving animal health across different geographical growing locations. However, global metabolome changes resulting from the growth environment are well-documented in the literature. For example, the study by Lee et al. (2010) [67] demonstrated that climatic factors, such as day length, temperature, and water availability, influence the green tea metabolome. Therefore, environmental variability in the accumulation of CTs and other metabolites in BFT could be exploited by plant breeders to enhance forage profiles for pasture bloat mitigation in ruminants. Additionally, this variability could aid in identifying geographically adapted BFT genotypes with optimized secondary metabolite accumulation.

## 3. Materials and Methods

### 3.1. Chemicals

Commercial standards used in this study were catechin (Cayman Chemical Company, Ann Arbor, MI, USA), epicatechin (Santa Cruz Biotechnology, Dallas, TX, USA), gallocatechin (Toronto Research Chemicals Inc., Vaughan, ON, Canada), epigallocatechin (HWI pharma services GmbH, Rülzheim, Germany), and procyanidins A2 (BOC Sciences, Shirley, NY, USA), B1 (BOC Sciences, Shirley, NY, USA), B2 (Santa Cruz Biotechnology, Dallas, TX, USA), B3 (Cayman Chemical Company, Ann Arbor, MI, USA), B4 (Cayman Chemical Company, Ann Arbor, MI, USA), and C1 (Cayman Chemical Company, Ann Arbor, MI, USA), and butanol, which were obtained from Sigma Aldrich (Oakville, ON, Canada). Chromatographic-grade acetone, methanol, and hydrochloric acid used for analysis were obtained from Thermo Fisher Scientific (Waltham, MA, USA). Double-distilled water was purified by the Milli-Q system.

### 3.2. Plant Material and Sample Collection

Eight BFT genotypes, BuUr-13, Leo, Norcen, Bruce, Pardee, NB95-118, Langille, and NB95-120, were used in this study. Seeds of BuUr-13, Leo, Norcen, Bruce, Pardee, and Langille were commercial cultivars, while NB95-118 and NB95-120 seeds were new breeder lines obtained from the birdsfoot trefoil breeding program in AAFC Kentville. All seeds planted in different geographical locations were taken from the same lot. Samples were harvested from BFT experimental plots in Kentville, Nova Scotia, Canada (45.0771° N, 64.4943° W), Logan, Utah, USA (41.6933° N, 111.8319° W), and Kingston, Rhode Island, USA (41.4896° N, 71.5352° W). This study was conducted in a randomized complete block design with four replications, and the plot sizes were 1.5 m × 3 m, established in August 2021 in Kentville, Nova Scotia, in June 2019 in Logan, Utah, and in September 2019 in Kingston, Rhode Island. BFT shoot samples, which included stems, leaves, and flowers, were collected from 2021, 2022, and 2023 in Kentville, while samples were collected across two years in Utah and Rhode Island in 2020 and 2021. To ensure that the powdered tissues used in the analysis were representative of the entire plots, multiple plants were sampled within a quadrat to account for potential intra-genotypic variability. These samples were pooled to create a composite sample, which was then homogenized to a fine powder for CT and metabolomic analysis. The sampled tissue consisted of the whole forage, including leaves, stems, and flowers, as these are the plant parts fed to animals. The detailed experimental plan and weather data from the sites and across years of sampling are described in the Supplementary Materials (Tables S1–S3). Due to a lack of rain in Utah, the experiment plots were irrigated beginning in mid-May once a week for four hours and received ~75 mm per week.

### 3.3. Condensed Tannin Analysis

Condensed tannins were quantified following the protocol of Grabber and Zeller (2020) [68], with some modifications. Ten milligrams (±2.00 mg) of freeze-dried and ground BFT samples was placed into a 15 mL disposable conical glass centrifuge tube (VWR, Mississauga, ON, Canada). An amount of 5 mL of extraction solution (70:30 acetone/water, *v*/*v*) was transferred into the tube enclosed with a polytetrafluoroethylene (PTFE)-faced screw cap and vortexed for 15 s. The tubes were then placed in a 1510 Branson sonicating water bath at 30 °C for 45 min. The tubes were removed every 15 min and vortexed. The samples were centrifuged at 3000× *g* for 5 min, after which 0.5 mL of the supernatant was transferred into a fresh 15 mL glass centrifuge tube and the remaining extraction solution decanted. To ensure complete extraction of the pellet, 0.5 mL of additional extraction solution was added to the pelleted plant material. Next, 4.5 mL of the reaction mixture (3.5 mM ammonium iron (III) sulfate dodecahydrate in butanol/acetone/HCl, 42:43:5) was added to both the tube containing the 0.5 mL of supernatant and the tube containing the remaining plant material pellet. The tubes were gently mixed, weighed, and placed on a heating block at 100 °C for exactly 45 min. Tubes were removed from the heating block, allowed to cool to room temperature, and reweighed to ensure no volume loss. The tubes were centrifuged for 5 min at 3000× *g*, and 200 µL was placed into a 96-well plate. Absorbances were recorded at 550 nm in duplicate using a Multiskan GO spectrophotometer (Thermo Scientific) for each of the three to four biological replicates. A calibration curve was constructed using CTs extracted from bulk BFT material using Sephadex LH-20 (Cytiva, Marlborough, MA, USA), following the method described by Hagerman and Butler (1991) [69].

### 3.4. LC-MS Metabolome Profiling

The metabolites of the BFT samples were extracted using 70% methanol as previously described by Yu et al. (2020) [70] with modifications. An amount of 1 mL of 70:30 methanol/water (*v*/*v*) was added to 50 mg (±2 mg) of ground plant material, vigorously vortexed for 15 s, and sonicated at room temperature for 30 min. The extract was centrifuged at 12,000× *g* for 10 min. The supernatant was transferred to a fresh microcentrifuge tube. A second extraction of the pellet was performed following the same procedure, and the supernatant was combined with the first extract. An amount of 1 mL of supernatant was filtered through a 0.22 µm polyvinylidene fluoride (PVDF) filter into amber glass HPLC vials for analysis. Extracts were analyzed using a Thermo Q-Exactive Orbitrap mass spectrometer coupled to an Agilent 1290 HPLC system (Santa Clara, CA, USA). A volume of 2 μL of each sample was injected onto a Zorbax Eclipse Plus RRHD C18 column (2.1 mm × 50 mm, 1.8 μm; Agilent) maintained at 35 °C using a flow rate of 0.3 mL min^−1^ with a mobile phase of LC–MS-grade water (Optima) with 0.1% formic acid (phase A) and LC–MS-grade acetonitrile (Optima) also with 0.1% formic acid (phase B). Mobile phase B was held at 2% for 45 sec before increasing to 22% over 0.5 min. Mobile phase B was increased to 35% over 1.75 min and to 100% over 3.5 min. Mobile phase B was maintained at 100% for 2.5 min before returning to 2% over 0.5 min. The following conditions were used for the positive Heated Electrospray Ionization (HESI): capillary voltage, 4.25 kV; capillary temperature, 400 °C; sheath gas, 30.0 units; auxiliary gas, 22 units; probe heater temperature, 450 °C; S-Lens RF level, 45.00. For metabolite profiling, three to four biological replicates were analyzed using a 140,000-resolution full-scan acquisition method across a 100–1250 *m*/*z* scan range, an automatic gain control (AGC) target of 5 × 10^5^, and a maximum injection time (maxIT) of 512 ms. For quality control, two composite samples were prepared, consisting of equal mixtures: QC1 (2021 samples) and QC2 (Kentville, Nova Scotia, 2021–2023). These were analyzed after every 27 samples. Additionally, for compound identification, composite samples were also analyzed by a data-dependent acquisition (DDA) method that consisted of a full scan at 35,000, an AGC of 3 × 10^6^, and a maxIT of 128 ms. The top 5 most intense ions of each full MS scan (dynamic exclusion: 10 s) were selected for MS/MS scans at 17,500 resolutions using normalized collision energy at 35 eV, an AGC of 5 × 10^6^, a maxIT of 64 ms, and an isolation window of 1.2 *m*/*z* units. For monitoring the instrument performance across the runs and measuring the retention indices of the detected compounds, N-alkyl pyridinium sulfonate retention index standards were also analyzed after every 54 samples [71].

The confidence levels in metabolite annotation were provided for all reported metabolites. Briefly, level 1 refers to identifications based on a feature comparison with reference standards, while level 2 refers to specific identification based on the accurate mass of the precursor ions and a comparison of fragmentation data to databases and/or the literature [71].

For targeted analysis, the peak areas of compounds with an identification confidence level of 1 or 2 were analyzed using the Thermo Tracefinder software package (version 5.2), allowing for a retention time variation of ≤0.05 min and a mass error of <5 ppm. For non-targeted analysis, “.raw” data files were converted to mzML files and centroided using Proteowizard [72]. Converted files were imported into R (4.4.0) to perform chromatogram alignment, feature detection, and peak extraction using the xcms package (3.2.0) [73]. Settings for XCMS processing were as follows: method, centWave [74]; prefilter, (5, 5000); ppm, 2.5; snthresh, 5; peakwidth, (5, 60); noise, 10,000,000; bw, 5; minfrac, 0.01; and mzwid, 0.015. Principal component analysis (PCA) of detected molecular features was performed with FactoMineR [75] and MetabolAnalyze [76].

### 3.5. Statistical Analysis

Statistical analysis was performed using two-way analysis of variance (ANOVA) in the R 4.4.0 statistical program. Data were checked for normality (Shapiro–Wilk test) and homogeneity of variance (Levene’s test) before the ANOVA. A post hoc test with least significant difference (LSD) was conducted to compare and separate the means of the groups. A *t*-test was conducted for the pairwise comparison of metabolites between locations, and the *p*-value was adjusted using the false discovery rate.

## Figures and Tables

**Table 1:** Analysis of variance (ANOVA) of extractable condensed tannins in eight birdsfoot trefoil genotypes from Kentville, Utah, and Rhode Island in 2021 and in three growth years in Kentville.

Source of Variation	df	F Statistic	*p* Value
Location by genotype in 2021			
Location	2	14.018	1.01 × 10^−5^ ***
Genotype	7	7.035	4.01 × 10^−6^ ***
Location × genotype	14	1.580	0.112
Residuals	60		
Year by genotype in Kentville			
Year	2	3.007	0.00569
Genotype	7	5.315	9.14 × 10^−54^ ***
Year × genotype	14	0.524	0.9096
Residuals	60		

*** Statistical significance at *p* < 0.001; df: degree of freedom. For Kentville in 2021, *n* = 3 for BuUr-13; *n* = 3 for Leo; *n* = 2 for Norcen; *n* = 4 for Bruce; *n* = 4 for Pardee; *n* = 4 for NB95-118; *n* = 4 for Langille; and *n* = 4 for NB95-120. For Kentville in 2022, *n* = 2 for BuUr-13; *n* = 4 for Leo; *n* = 3 for Norcen; *n* = 4 for Bruce; *n* = 2 for Pardee; *n* = 3 for NB95-118; *n* = 3 for Langille; and *n* = 4 for NB95-120. For Kentville in 2023, *n* = 3 for BuUr-13; *n* = 4 for Leo; *n* = 4 for Norcen; *n* = 4 for Bruce; *n* = 4 for Pardee; *n* = 4 for NB95-118; *n* = 4 for Langille; and *n* = 4 for NB95-120. For Utah in 2021, *n* = 4 for BuUr-13 Leo, Norcen, Bruce, Pardee, NB95-118, Langille, and NB95-120. For Rhode Island in 2021, *n* = 3 for BuUr-13, Leo, Norcen, Bruce, Pardee, NB95-118, Langille, and NB95-120.

**Table 2:** Major compounds detected in trefoil genotypes using LC-MS.

#	Compound Name	Formula	NAPS RI	Ion Type	*m*/*z*	Mass Error (ppm)	rt (min)	Confidence
1	Tryptophan	C_11_H_12_N_2_O_2_	493	[M+H]^+^	205.0972	0.22	2.32	1
2	Rutin	C_27_H_30_O_16_	528	[M+H]^+^	611.1606	−0.13	2.52	2
3	Kaempferol 3-rhamnoside-7-glucoside	C_27_H_30_O_15_	548	[M+H]^+^	595.1657	−0.06	2.63	2
4	Kaempferitrin	C_27_H_30_O_14_	584	[M+H]^+^	579.1709	0.01	2.83	1
5	Coumaric acid	C_9_H_8_O_3_	612	[M+H]^+^	165.0547	0.09	2.99	2
6	Kaempferol 7-O-rhamnoside	C_21_H_20_O_10_	642	[M+H]^+^	433.1128	−0.31	3.16	2
7	Soyasaponin I	C_48_H_78_O_18_	1003	[M+H]^+^	943.5254	−0.74	5.77	2
8	Soyasaponin βg	C_54_H_84_O_21_	1087	[M+H]^+^	1069.5570	−0.73	6.08	2
9	Glucosyl-monolinolein	C_27_H_46_O_9_	1358	[M+Na]^+^	537.3040	1.03	6.97	2
10	Diglucosyl-monolinolein	C_33_H_56_O_14_	1185	[M+Na]^+^	699.3565	0.32	6.47	2
11	Caffeic acid	C_9_H_8_O_4_	1022	[M+H]^+^	181.0494	−0.86	5.84	2
12	Ferulic acid	C_10_H_10_O_4_	660	[M+H]^+^	195.0651	−0.49	3.26	2
13	Cyanidin	C_15_H_11_O_6_	625	[M+]	287.0548	−0.61	3.06	2
14	Catechin	C_15_H_14_O_6_	518	[M+H]^+^	291.0863	−0.05	2.46	1
15	Riboflavin	C_17_H_20_N_4_O_6_	526	[M+H]^+^	377.1455	−0.06	2.51	2
16	Kaempferol 3-glucoside	C_21_H_20_O_11_	641	[M+H]^+^	449.1076	−0.6	3.15	2
17	Cyanidin 3-O-glucoside	C_21_H_21_O_11_	555	[M+]	449.1079	0.16	2.67	2
18	Corniculatusin 3-glucoside	C_22_H_22_O_13_	589	[M+H]^+^	495.1133	−0.07	2.86	2
19	Phosphatidylcholine lyso 18:2	C_26_H_50_NO_7_P	1310	[M+H]^+^	520.3402	0.74	6.82	2
20	Procyanidin B1	C_30_H_26_O_12_	495	[M+H]^+^	579.1501	0.67	2.33	1
21	Sexangularetin 3-rhamnoside-7-glucoside	C_28_H_32_O_16_	555	[M+H]^+^	625.1764	0.13	2.67	2
22	Kaempferol-malonyl-rhamnose-rhamnose	C_30_H_32_O_17_	644	[M+H]^+^	665.1707	−0.76	3.17	2
23	Kaempferol 3-rutinoside-7-rhamnoside	C_33_H_40_O_19_	511	[M+H]^+^	741.2235	−0.2	2.42	2
24	PC(C16:0/C18:3)	C_42_H_78_NO_8_P	1611	[M+]	756.5537	−0.17	7.74	2
25	Kaempferol-glucosyl-rhamnosyl-glucoside	C_33_H_40_O_20_	504	[M+H]^+^	757.2178	−0.98	2.38	2

rt: retention time; *m*/*z*: mass-to-charge ratio; identification confidence [62]: 1 = matching standard, and 2 = high MS/MS spectra library match.

**Table 3:** Targeted identification of monomeric and low-molecular-weight CT compounds in BFT genotypes using LC-MS.

#	Compound Name	Formula	NAPS RI	Ion Type	*m*/*z*	Mass Error (ppm)	rt (min)	Confidence
1	Catechin	C_15_H_14_O_6_	518	[M+H]^+^	291.0863	−0.05	2.46	1
2	Epicatechin	C_15_H_14_O_6_	541	[M+H]^+^	291.08664	1.12	2.59	1
3	Gallocatechin	C_15_H_14_O_7_	476	[M+H]^+^	307.08139	0.52	2.22	1
4	Epigallocatechin	C_15_H_14_O_7_	500	[M+H]^+^	307.08137	0.45	2.35	1
5	Procyanidin B1	C_30_H_26_O_12_	495	[M+H]^+^	579.15009	0.67	2.33	1
6	Procyanidin B4	C_30_H_26_O_12_	521	[M+H]^+^	579.1499	0.34	2.48	1
7	Procyanidin trimer-1	C_45_H_38_O_18_	512	[M+H]^+^	867.21338	0.33	2.43	2
8	Procyanidin trimer-2	C_45_H_38_O_18_	541	[M+H]^+^	867.2135	0.47	2.59	2
9	Procyanidin trimer-3	C_45_H_38_O_18_	463	[M+H]^+^	867.2136	0.59	2.15	3
10	Prodelphinidin trimer	C_45_H_38_O_20_	482	[M+H]^+^	899.2028	−0.11	2.26	2

rt: retention time; *m*/*z*: mass-to-charge ratio; identification confidence [62]: 1 = matching standard, 2 = high MS/MS spectra library match, and 3 = MS1 *m*/*z* database match.

## Data Availability

The original contributions presented in this study are included in the article/supplementary material. Further inquiries can be directed to the corresponding authors.

## Supplementary Materials

The following supporting information can be downloaded at https://www.mdpi.com/article/10.3390/plants14172766/s1, Table S1. Experimental design for birdsfoot trefoil field trials at three locations; Table S2. Mean monthly temperature (°C) from growing locations and across years of sampling; Table S3. Total monthly precipitation (mm) from growing locations and across seasons of sampling; Figure S1. Soluble condensed tannin content in the eight birdsfoot trefoil cultivars in (a) Rhode Island (2020 and 2021) and (b) Utah (2020 and 2021). (c) Boxplot comparing extractable CT levels between Kentville, Rhode Island, and Utah in 2021. (d) Boxplot comparing extractable CT levels within Kentville (in 2021, 2022, and 2023), Rhode Island (in 2020 and 2021), and Utah (in 2020 and 2021). Extractable condensed tannins were quantified by the butanol–HCl method in mg/g dried weight of BFT samples. Data in bar plots are shown as the mean ± standard error of the mean (for *n* = 3–4 biologically independent replicates); the different letters above the bars represent statistical differences determined by two-way analysis of variance (ANOVA; columns followed by a different letter are different, *p* < 0.05) using R 4.4.0; Table S4. Analysis of variance of insoluble condensed tannins in eight birdsfoot trefoil cultivars from Kentville, Utah, and Rhode Island in 2021 and three growth years in Kentville; Figure S2. Insoluble condensed tannin contents in the eight birdsfoot trefoil cultivars across (a) three locations and (b) three growing seasons. Extractable condensed tannins were quantified by the butanol–HCl method in mg/g dried weight of BFT samples in Kentville (in 2021, 2022, and 2023), Rhode Island, and Utah (in 2020 and 2021). Data are shown as the mean ± standard error of the mean (for *n* = 3–4 biologically independent replicates); the different letters above the bars represent statistical differences determined by two-way analysis of variance (ANOVA; columns followed by a different letter are different, *p* < 0.05) using R 4.4.0; Figure S3. Insoluble condensed tannin contents in the eight birdsfoot trefoil cultivars in (a) Rhode Island (2020 and 2021) and (b) Utah (2020 and 2021). (c) Boxplot comparing insoluble CT levels between Kentville, Rhode Island, and Utah in 2021. (d) Boxplot comparing insoluble CT levels within Kentville (in 2021, 2022, and 2023), Rhode Island (in 2020 and 2021), and Utah (in 2020 and 2021). Insoluble condensed tannins were quantified by the butanol–HCl method in mg/g dried weight of BFT samples. Data in bar plots are shown as the mean ± standard error of the mean (for *n* = 3–4 biologically independent replicates); the different letters above the bars represent statistical differences determined by two-way analysis of variance (ANOVA; columns followed by a different letter are different, *p* < 0.05) using R 4.4.0; Figure S4. Heatmap of CT-related monomeric compounds and low-molecular-weight procyanidins from three growth locations in 2021. The scaled values in the heatmap are the average z-scores of the peak areas extracted from the LC-MS analysis of the BFT samples. The red in the heatmap indicates higher metabolite accumulation across locations, while blue indicates lower metabolite accumulation across locations; Figure S5. Differentially accumulated metabolites of BFT between (a) Kentville and Rhode Island, (b) Utah and Rhode Island, and (c) Kentville and Utah. (d) Number of differentially accumulated metabolites between the pairwise comparison of three growth locations in 2021. Color: Green indicates higher metabolite accumulation in the latter, while red indicates lower metabolite accumulation in the latter, and vice versa; Figure S6. Differentially accumulated metabolites of BFT between (a) 2021 and 2022, (b) 2021 and 2023, and (c) 2022 and 2023. (d) Number of differentially accumulated metabolites between the pairwise comparison of 2021, 2022, and 2023 in Kentville. Color: Green indicates higher metabolite accumulation in the latter, while red indicates lower metabolite accumulation in the latter, and vice versa; Table S5. List of specific detected compounds from BFT metabolomics; Figure S7. Fragmentation pathway of (a) catechin and larger molecules, (b) procyanidin B2, and (c) procyanidin B4 generates a diagnostic product ion at *m*/*z* 139.0392 (C_7_H_8_O_3_^+^) which corresponds to the A ring of catechin. Identical product ions are observed for epicatechin (Table S5); Figure S8. MS/MS of the following aglycones: (a) Kaempferol, the most abundant flavanol detected in the samples, shows limited fragmentation at the selected normalized collision energy. In contrast, kaempferol conjugates, including (b) kaempferol 7-O-rhamnoside, (c) kaempferitrin, and (d) kaempferol 3-rhamnoside-7-glucoside, show facile neutral loss of the glycoside groups to generate a kaempferol diagnostic product ion. This product ion indicates that the conjugated molecule is based on kaempferol; Figure S9. MS/MS of (a) soyasaponin I; (b) soyasaponin βg; (c) soyasaponin γg; (d) dehydrosoyasaponin I. The product ions at *m*/*z* 423 are derived from the core constituent soyasapogenol B following loss of 2H_2_O. The diagnostic product ion of (d) dehydrosoyasaponin I is 2 mass units lower, since the core constituent is dehydrosoyasapogenol B, which contains a keto group at C-22 in place of the hydroxy group of soyasapogenol B.

## Abbreviations

The following abbreviations are used in this manuscript:

AAFC	Agriculture and Agri-Food Canada
AGC	Automatic gain control
ANOVA	Analysis of variance
ANR	Anthocyanidin reductase
BFT	Birdsfoot trefoil
CTs	Condensed tannins
DAM	Differentially accumulated metabolite
DDA	Data-dependent acquisition
HESI	Heated electrospray ionization
LAR	Leucoanthocyanidin reductase
LC-MS	Liquid chromatography–mass spectrometry
LSD	Least significant difference
MW	Molecular weight
PCs	Procyanidins
PC1	Principal component 1
PC2	Principal component 2
PCA	Principal component analysis
PDs	Prodelphinidins
