# Supplementary material for: Profiling Environmental Variations in Condensed Tannins and Other Metabolites of Birdsfoot Trefoil (Lotus corniculatus L.) Genotypes
Source: Plants (Basel). 2025 Sep 4;14(17):2766. doi: 10.3390/plants14172766 (PMC12430702; doi:10.3390/plants14172766)
Supplement: Supplementary file 1 [file plants-14-02766-s001.zip › plants-3808067-supplementary.pdf]

Profiling environmental variations in condensed tannins and other metabolites of birdsfoot trefoil (*Lotus corniculatus* L.) genotypes

Solihu Kayode Sakariyahu [1,2,3], Tim McDowell [1], Justin B. Renaud [1,*], Yousef Papadopoulos [4], Kathleen Glover [4], Rebecca Nelson Brown [5], Michael D. Peel [6], Heathcliffe Riday [7], Susanne E. Kohalmi [2] and Abdelali Hannoufa [1,2,*]

[1] London Research and Development Center, Agriculture and Agri-Food Canada, London, ON N5V 4T3, Canada; solihukayode.sakariyahu@agr.gc.ca (S.K.S.); tim.mcdowell@agr.gc.ca (T.M.)

[2] Department of Biology, University of Western Ontario, London, ON N6A 3K7, Canada; skohalmi@uwo.ca

[3] Department of Biology, Ahmadu Bello University, Zaria 810107, Nigeria

[4] Kentville Research and Development Centre, Agriculture and Agri-Food Canada, Kentville, NS B4N 1J5, Canada; yousef.papadopoulos@agr.gc.ca (Y.P.); kathleen.glover@agr.gc.ca (K.G.)

[5] Department of Plant Sciences and Entomology, University of Rhode Island, Kingston, RI 02881, USA; brownreb@uri.edu

[6] USDA-ARS Forage and Range Research Laboratory, Utah State University, Logan, UT 84321, USA; mike.peel@ars.usda.gov

[7] USDA-ARS US Dairy Forage Research Center, Madison, WI 53706, USA; heathcliffe.riday@usda.gov

* Correspondence: justin.renaud@agr.gc.ca (J.B.R.); abdelali.hannoufa@agr.gc.ca (A.H.)

Supporting Information

Table S1. Experimental design for birdsfoot trefoil field trials in three locations

Experimental activities	Kentville	Utah	Rhode Island
Coordinates of field site	45.0771°N, 64.4943°W	41.6933°N, 111.8319°W	41.4896°N, 71.5352°W
Soil type	Coarse loamy mixed with nonclay	Nibley silty clay loam	Bridgehampton Silt loam
Elevation	31 m	1381 m	30 m
Experimental design	RCBD	RCB latinized 2 row × 4 column	RCBD
Plot size	1.5m × 3m	1.5m × 3m	1.5m × 3m
Planting date	19-May-2021	02-June-2019	06-September-2019
Harvest date	03-August-2021	25-August-2020	24-June-2020
	21-July-2022	25-August-2021	23-June-2021
	19-July-2023		
Method of harvest	Plants within quadrat of 60 cm by 25 cm (0.15 m^2 area) were harvested.	Plants within quadrat of 60 cm by 25 cm (0.15 m^2 area) were harvested cleaned of weed at 10-15 cm height	Plants within quadrat of 36 cm by 36 cm (0.135 m^2 area) were harvested.
Number of replicates	4	4	3

Table S2. Mean monthly temperature (°C) from growing locations and across years of sampling

**Month**	**Kentville**			**Utah**		**Rhode Island**	
	**2021**	**2022**	**2023**	**2020**	**2021**	**2020**	**2021**
January	-2.7	-5.8	-0.4	-2.4	-3.7	1.5	-0.6
February	-3.0	-3.4	-5.6	-3.7	-0.5	1.8	-0.9
March	0.5	1.2	-0.3	4.3	3.0	5.3	3.9
April	6.3	6.1	6.4	7.7	7.2	6.0	8.5
May	10.8	11.9	10.1	13.5	12.8	12.9	13.4
June	18.5	16.2	15.9	16.3	22.2	19.2	19.9
July	18.9	20.9	22.3	21.1	24.6	23.2	21.2
August	20.4	21.0	18.6	22.0	19.6	22.4	22.4
September	16.7	15.7	16.9	14.6	15.2	17.3	18.4
October	11.1	11.4	11.7	8.2	7.9	12.1	14.1
November	4.6	5.6	2.4	1.8	3.5	8.1	5.3
December	-0.1	1.2	1.1	-5.5	-1.6	1.5	3.4
**Mean**	**8.5**	**8.5**	**8.3**	**8.1**	**9.2**	**10.9**	**10.8**

Table S3. Total monthly precipitation (mm) from growing locations and across seasons of sampling

Month	Kentville			*Utah		Rhode Island	
	2021	2022	2023	2020	2021	2020	2021
January	56.4	163.3	226.4	19.6	16.3	38.4	56.0
February	137.2	163.1	48.9	9.4	28.7	89.3	116.2
March	50.0	93.4	90.3	50.8	22.6	149.6	82.4
April	98.4	87.3	45.3	11.9	30.2	132.2	91.5
May	108.9	38.2	31.2	15.8	10.4	87.9	111.2
June	32.8	94.9	238.6	63.5	0.0	54.2	34.2
July	164.3	36.8	132.8	3.8	10.2	50.3	117.4
August	121.7	120.5	164.6	46.2	46.5	28.7	156.4
September	168.7	94.3	180.7	3.1	13.7	23.9	206.5
October	64.4	73.1	119.4	9.4	102.1	155.7	116.4
November	136.9	74.5	74.6	19.1	14.5	194	47.6
December	93.5	129	116.3	12.5	31.8	195.6	44.8
**Total**	**1233.2**	**1168.4**	**1469.1**	**264.9**	**326.9**	**1199.8**	**1180.6**

*Supplemented with irrigation in mid-May once a week for four hours and received ~ 75 mm per week.

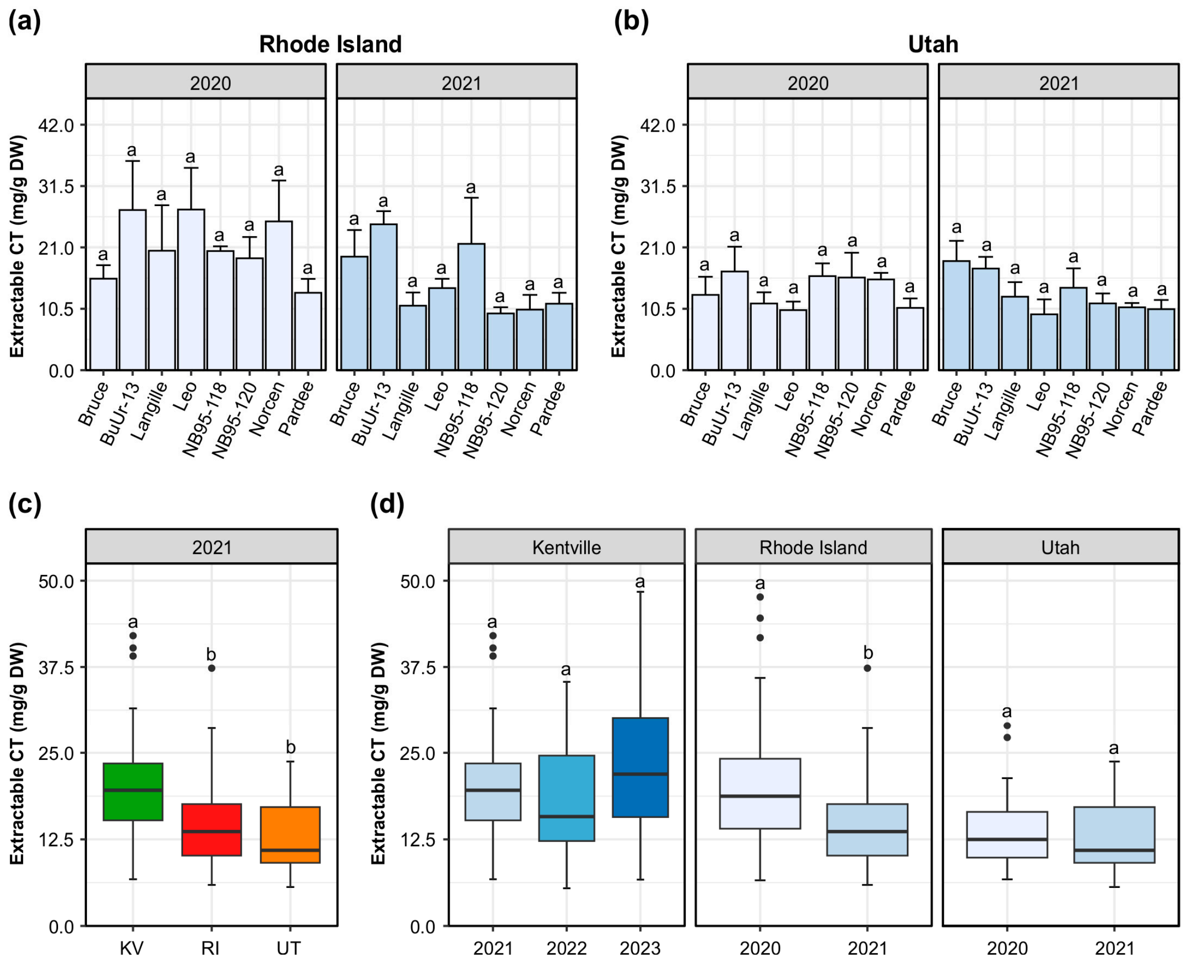

Figure S1. The soluble condensed tannins content in the eight birdsfoot trefoil cultivars in **(a)** Rhode Island (2020 and 2021) and **(b)** Utah (2020 and 2021). **(c)** The boxplot comparing extractable CT levels between Kentville (KV), Rhode Island (RI) and Utah (UT) in 2021. **(d)** The boxplot comparing extractable CT levels within Kentville (in 2021, 2022 and 2023), Rhode Island (in 2020, and 2021) and Utah (in 2020, and 2021). Extractable condensed tannins were quantified by the butanol-HCl method in mg/g dried weight of BFT samples. Data in bar plots are shown as the mean ± standard error of the mean (for n = 3 – 4 biologically independent replicates); the different letters above the bars represent statistical differences determined by two-way analysis of variance (ANOVA; columns followed by a different letter are different, $p<0.05$) using R 4.4.0.

Table S4. Analysis of variance of insoluble condensed tannins in eight birdsfoot trefoil cultivars from Kentville, Utah and Rhode Island in 2021 and three growth years in Kentville.

**Source of variation**	**df**	**F statistic**	***p* value**
Location by cultivar in 2021			
Location	2	17.767	8.71e-07 ***
Genotype	7	5.010	0.000163 ***
Location × genotype	14	1.256	0.261903
Residuals	60		
Genotype by year in Kentville			
Year	2	5.500	6.44e-05 ***
Genotype	7	62.454	2.17e-15 ***
Year × genotype	14	0.998	0.467
Residuals	60		

*** Statistical significance at $p < 0.001$
df: degree of freedom

For Kentville in 2021, n = 3 for BuUr-13, n = 3 for Leo, n = 2 for Norcen, n = 4 for Bruce, n = 4 for Pardee, n = 4 for NB95-118, n = 4 for Langille, and n = 4 for NB95-120.

For Kentville in 2022, n = 2 for BuUr-13, n = 4 for Leo, n = 3 for Norcen, n = 4 for Bruce, n = 2 for Pardee, n = 3 for NB95-118, n = 3 for Langille, and n = 4 for NB95-120.

For Kentville in 2023, n = 3 for BuUr-13, n = 4 for Leo, n = 4 for Norcen, n = 4 for Bruce, n = 4 for Pardee, n = 4 for NB95-118, n = 4 for Langille, and n = 4 for NB95-120.

For Utah in 2021, n = 4 for BuUr-13, Leo, Norcen, Bruce, Pardee, NB95-118, Langille, and NB95-120.

For Rhode Island in 2021, n = 3 for BuUr-13, Leo, Norcen, Bruce, Pardee, NB95-118, Langille, and NB95-120.

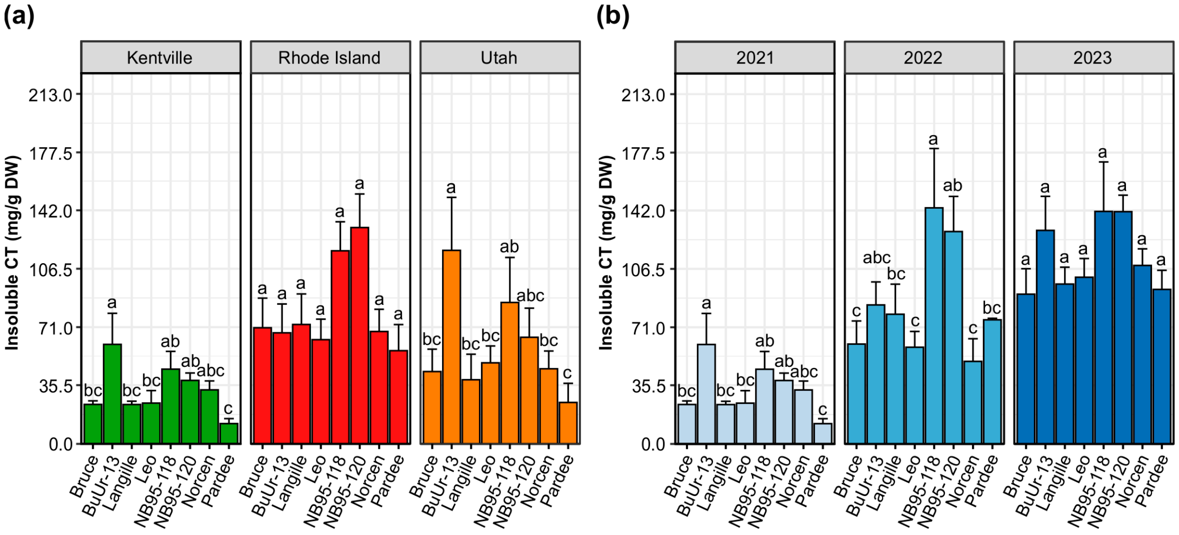

Figure S2. The insoluble condensed tannins content in the eight birdsfoot trefoil cultivars across **(a)** three locations and **(b)** three growing seasons. Extractable condensed tannins were quantified by the butanol-HCl method in mg/g dried weight of BFT samples in Kentville (in 2021, 2022, and 2023), Rhode Island and Utah (in 2020 and 2021). Data are shown as the mean ± standard error of the mean (for n = 3 – 4 biologically independent replicates); the different letters above the bars represent statistical differences determined by two-way analysis of variance (ANOVA; columns followed by a different letter are different, $p<0.05$) using R 4.4.0.

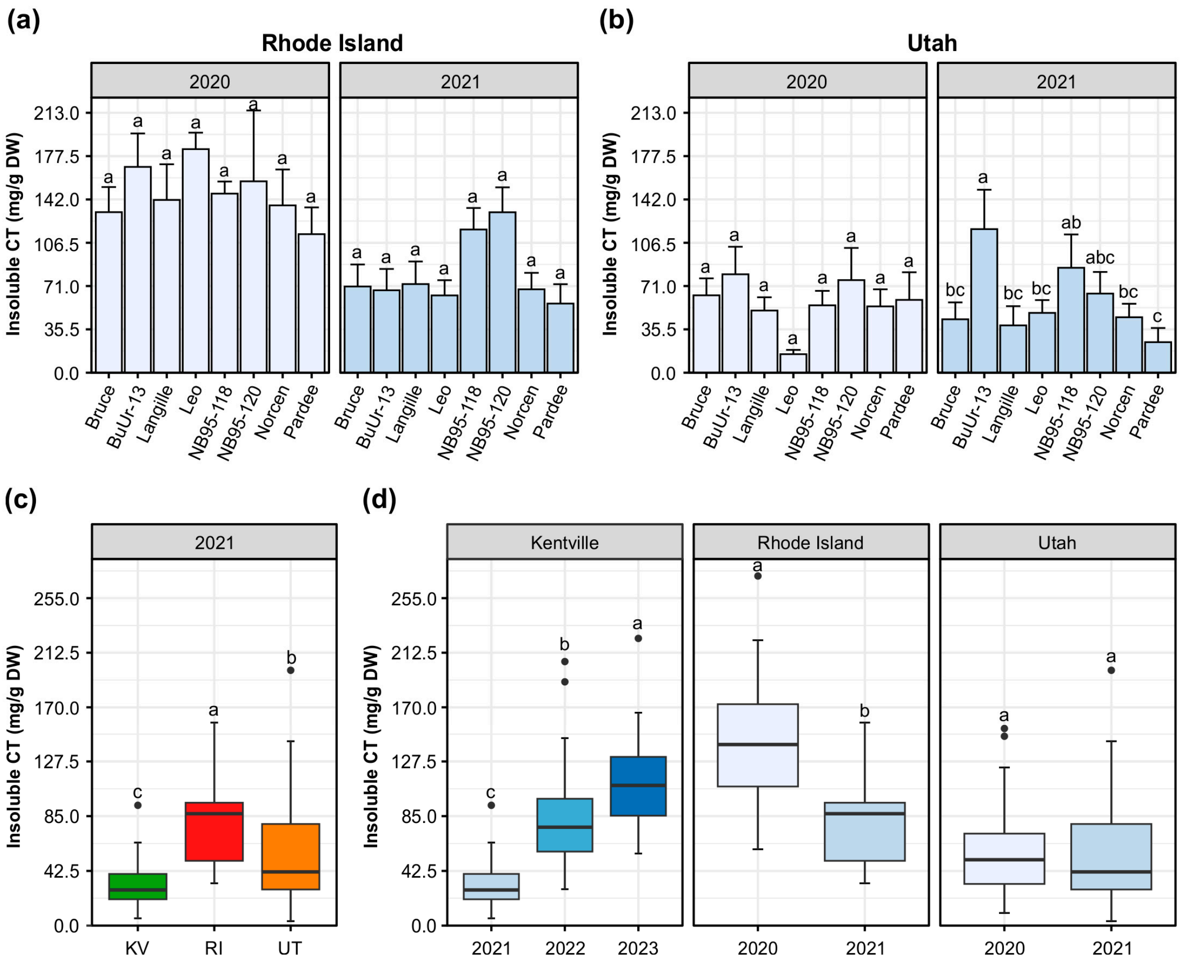

Figure S3. The insoluble condensed tannins content in the eight birdsfoot trefoil cultivars in **(a)** Rhode Island (2020 and 2021) and **(b)** Utah (2020 and 2021). **(c)** The boxplot comparing insoluble CT levels between Kentville (KV), Rhode Island (RI) and Utah (UT) in 2021. **(d)** The boxplot comparing insoluble CT levels within Kentville (in 2021, 2022 and 2023), Rhode Island (in 2020, and 2021) and Utah (in 2020, and 2021). Insoluble condensed tannins were quantified by the butanol-HCl method in mg/g dried weight of BFT samples. Data in bar plots are shown as the mean ± standard error of the mean (for n = 3 – 4 biologically independent replicates); the different letters above the bars represent statistical differences determined by two-way analysis of variance (ANOVA; columns followed by a different letter are different, p<0.05) using R 4.4.0.

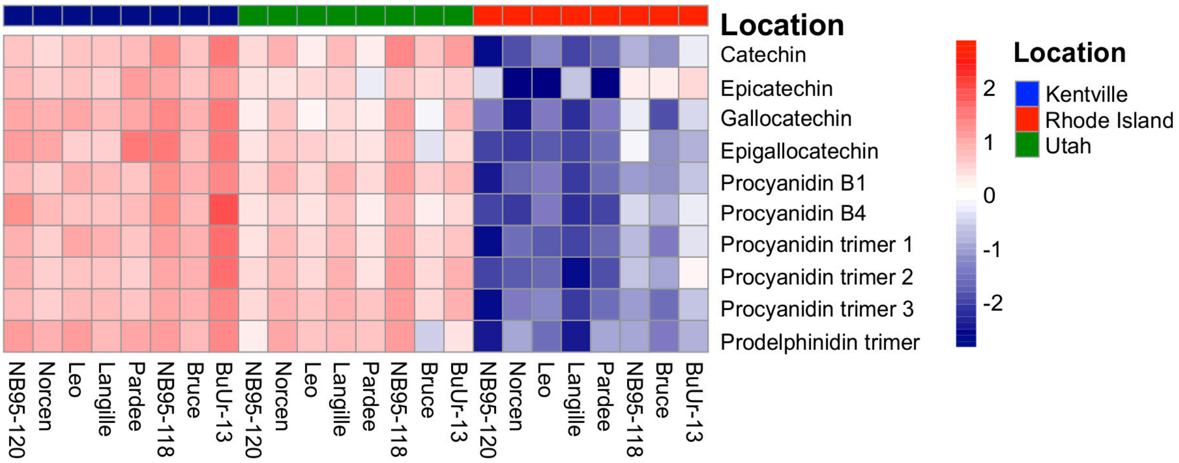

Figure S4. Heatmap of CT-related monomeric compounds and low molecular weight procyanidins from three growth locations in 2021. The scaled values in the heatmap are the average z-score of the peak areas extracted from LC-MS analysis of BFT samples. The red in the heatmap indicates higher, while blue indicates lower metabolite accumulation across locations.

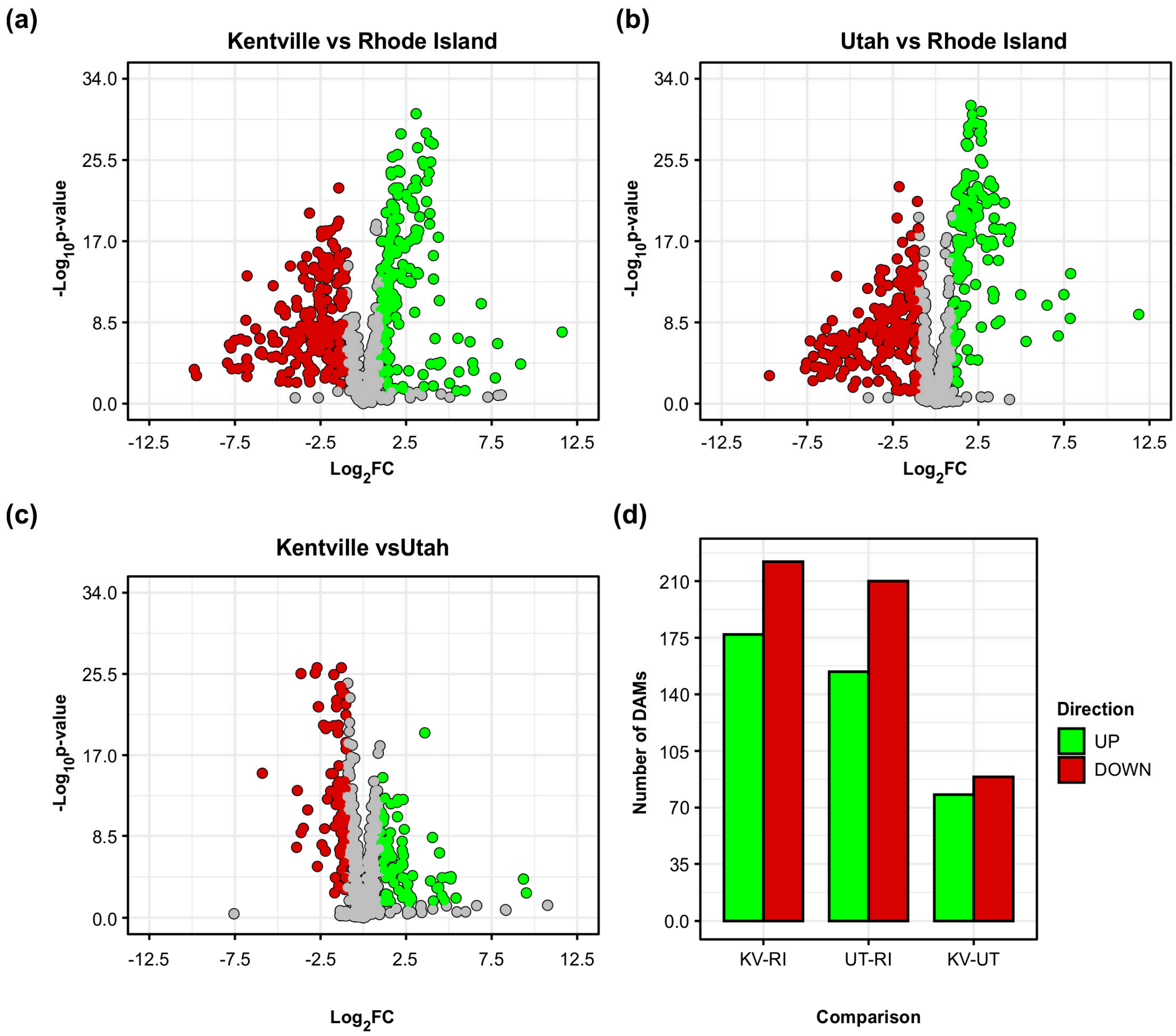

Figure S5. Differentially accumulated metabolites of BFT between **(a)** Kentville vs Rhode Island, **(b)** Utah vs Rhode Island and **(c)** Kentville vs Utah. **(d)** Number of differentially accumulated metabolites (DAMs) between the pairwise comparison of three growth locations in 2021. KV = Kentville, RI = Rhode Island and UT = Utah. Colour: green indicates the higher metabolites accumulated in the latter, while red indicates the lower metabolites accumulated in the latter and *vice versa*.

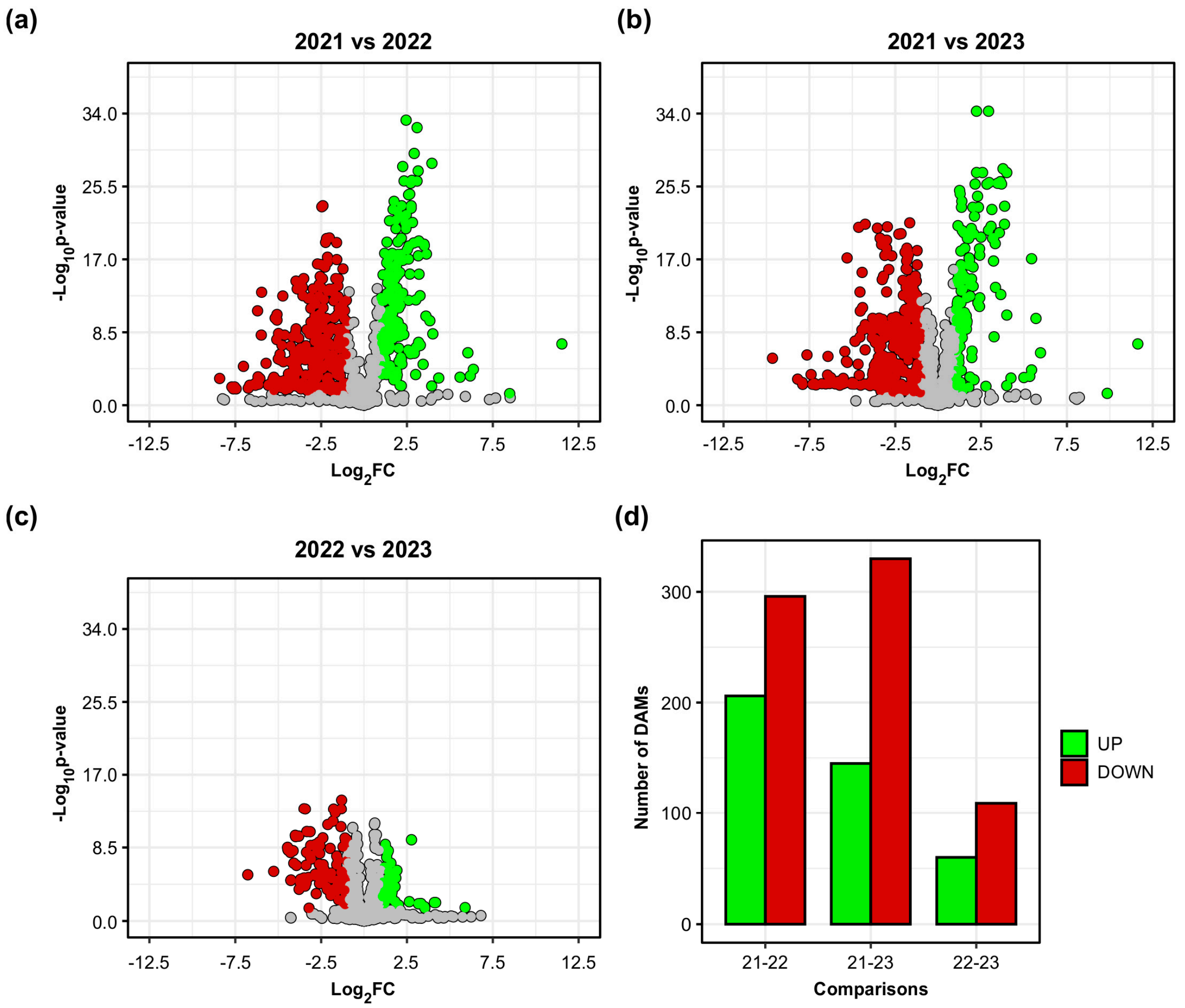

Figure S6. Differentially accumulated metabolites of BFT between **(a)** 2021 vs 2022, **(b)** 2021 vs 2023 and **(c)** 2022 vs 2023. **(d)** Number of differentially accumulated metabolites between the pairwise comparison of 2021, 2022 and 2023 in Kentville. Colour: green indicates the higher metabolites accumulated in the latter, while red indicates the lower metabolites accumulated in the latter and *vice versa*.

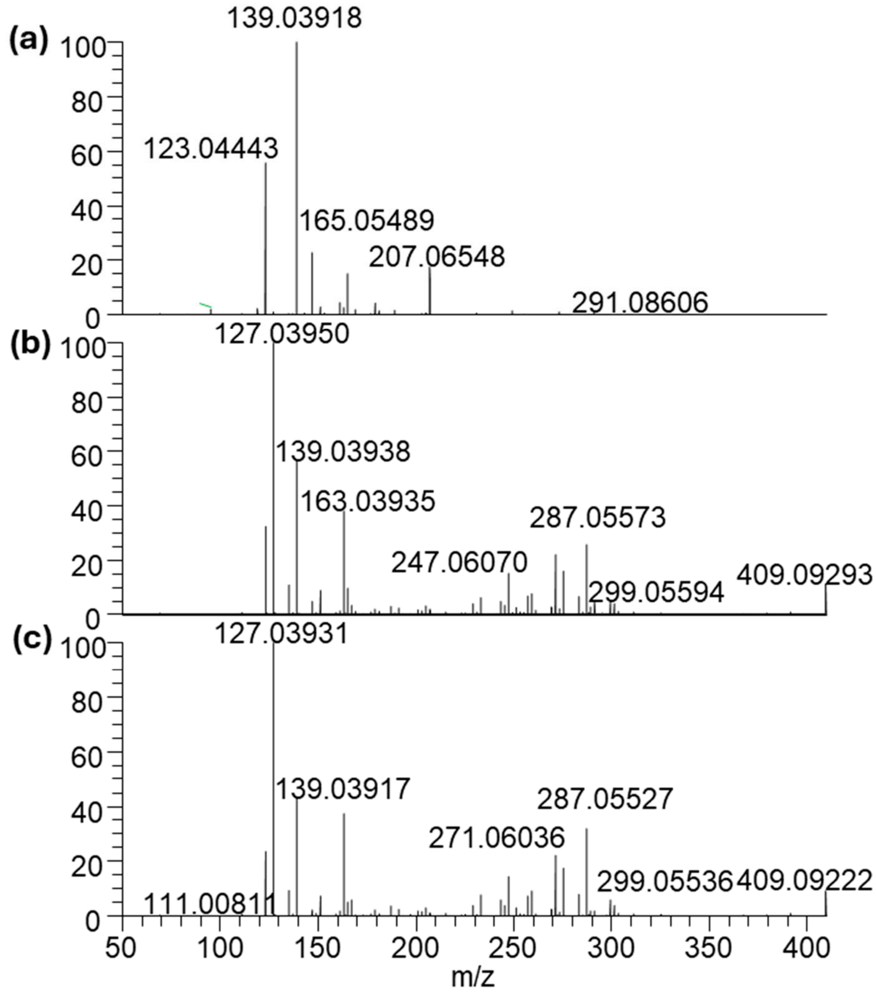

Figure S7. The fragmentation pathway of (**a**) catechin and larger molecules (**b**) Procyanidin B2 and (**c**) Procyanidin B4, generates a diagnostic product ion at *m/z* 139.0392 ($C_7H_8O_3^+$) which corresponds to the A ring of catechin. Identical product ions are observed for epicatechin (Table S5).

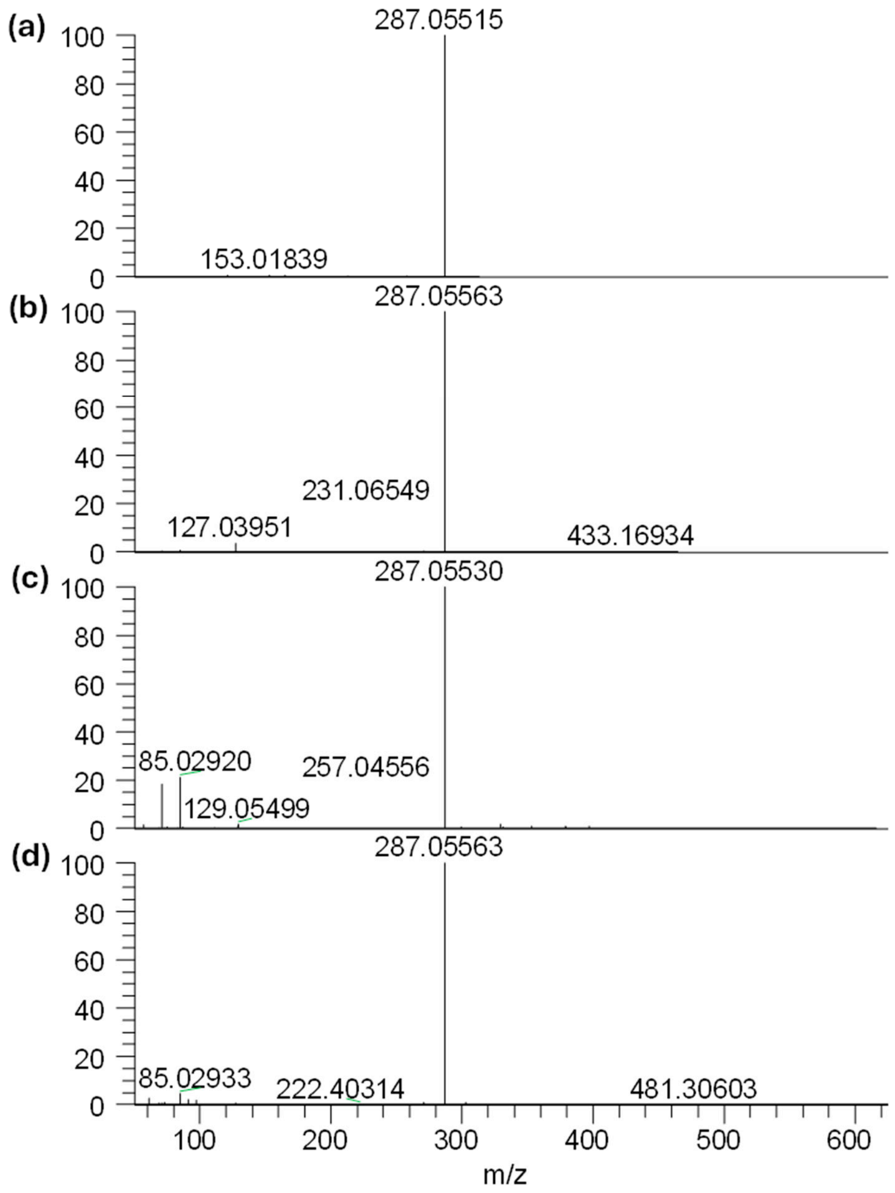

Figure S8. MS/MS of the aglycone (**a**) Kaempferol, the most abundant flavanol detected in the samples shows limited fragmentation at the selected normalized collision energy. In contrast, Kaempferol conjugates, including (**b**) Kaempferol 7-O-rhamnoside, (**c**) Kaempferitrin, and (**d**) Kaempferol 3-rhamnoside-7-glucoside show facile neutral loss of the glycoside groups to generate a kaempferol diagnostic product ion. This product ion is indicative that the conjugated molecule is based on kaempferol.

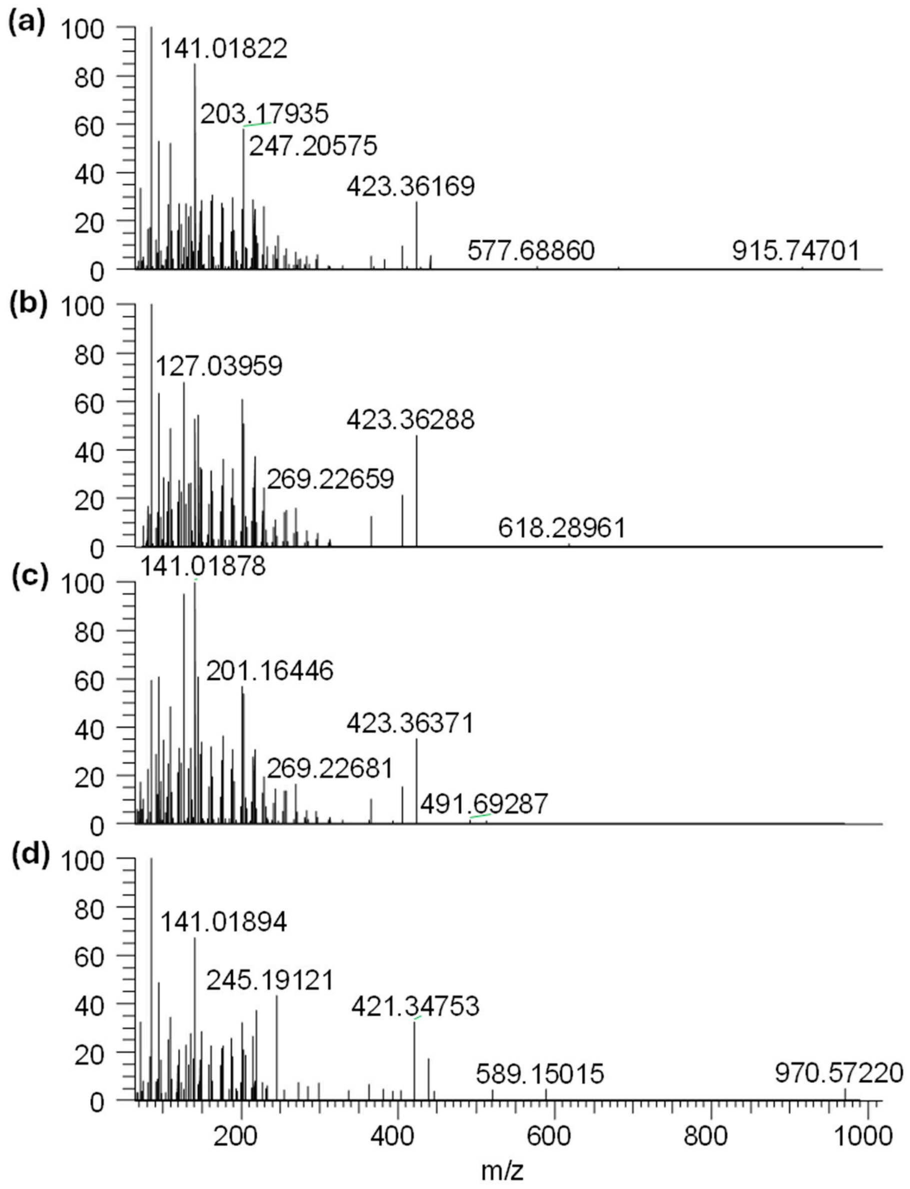

Figure S9. MS/MS of (**a**) Soyasaponin I, (**b**) Soyasaponin βg (**c**) Soyasaponin γg, (**d**) Dehydrosoyasaponin I. The product ions at *m/z* 423 are derived from the core constituent soyasapogenol B following loss of 2 H_2O. The diagnostic product ion of (d) Dehydrosoyasaponin I is 2 mass units lower, since the core constituent is dehydrosoyasapogenol B, which contains a keto group at C-22 in place of the hydroxy group of soyasapogenol B.

Table S5. The list of specific detected compounds from BFT metabolomics.

#	Compound Name	Formula	NAPS RI	Ion type	*m/z*	mass error (ppm)	Product Ions (*m/z*)	rt (min)	Confidence
1	Tryptophan	$C_{11}H_{12}N_2O_2$	493	$[M+H]^+$	205.0972	0.22	186.0711, 146.0604	2.32	1
2	Rutin	$C_{27}H_{30}O_{16}$	528	$[M+H]^+$	611.1606	-0.13	303.0507	2.52	2
3	Kaempferol 3-rhamnoside-7-glucoside	$C_{27}H_{30}O_{15}$	548	$[M+H]^+$	595.1657	-0.06	287.0551	2.63	2
4	Kaempferitrin	$C_{27}H_{30}O_{14}$	584	$[M+H]^+$	579.1708	0.01	287.0553	2.83	2
5	Coumaric acid	$C_9H_8O_3$	612	$[M+H]^+$	165.0547	0.3	147.0444, 119.0497	2.99	3
6	Kaempferol 7-O-rhamnoside	$C_{21}H_{20}O_{10}$	642	$[M+H]^+$	433.1128	-0.31	287.0555	3.16	2
7	Soyasaponin I	$C_{48}H_{78}O_{18}$	1003	$[M+H]^+$	943.5254	-0.74	423.3628, 85.0292	5.77	2
8	Soyasaponin βg	$C_{54}H_{84}O_{21}$	1087	$[M+H]^+$	1069.557	-0.73	423.36295, 85.0292	6.08	2
9	Glucosyl-monolinolein	$C_{27}H_{46}O_9$	1358	$[M+Na]^+$	537.304	1.03	185.0417	6.97	2
10	Diglucosyl-monolinolein	$C_{33}H_{56}O_{14}$	1185	$[M+Na]^+$	699.3565	0.32	347.0948	6.47	2
11	Caffeic acid	$C_9H_8O_4$	1022	$[M+H]^+$	181.0494	-0.86	135.1171, 163.1119	5.84	2
12	Ferulic Acid	$C_{10}H_{10}O_4$	660	$[M+H]^+$	195.0651	-0.49	177.0551, 145.0288	3.26	2
13	Cyanidin	$C_{15}H_{11}O_6$	625	[M+]	287.0548	-0.61	153.0186, 121.0289	3.06	1
14	Kaempferol	$C_{15}H_{10}O_6$	888	$[M+H]^+$	287.0549	-0.5	153.0186, 165.0188	5.26	1
15	Catechin	$C_{15}H_{14}O_6$	518	$[M+H]^+$	291.0863	-0.05	139.0391, 123.0444	2.46	1
16	Epicatechin	$C_{15}H_{14}O_6$	541	$[M+H]^+$	291.0866	1.12	139.0393, 123.0444	2.59	1
17	Quercetin	$C_{15}H_{10}O_7$	802	[M+]	303.0499	-0.1	257.0446, 229.0506	4.39	1

18	Gallocatechin	$C_{15}H_{14}O_7$	476	$[M+H]^+$	307.0814	0.52	215.1392, 243.1704	2.22	1
19	Riboflavin	$C_{17}H_{20}N_4O_6$	526	$[M+H]^+$	377.1455	-0.06	243.0877, 359.1348	2.51	2
20	Kaempferol 3-glucoside	$C_{21}H_{20}O_{11}$	641	$[M+H]^+$	449.1076	-0.6	287.0558	3.15	2
21	Cyanidin 3-O-glucoside	$C_{21}H_{21}O_{11}$	555	[M+]	449.1079	0.16	303.0498	2.67	2
22	Catechin-3'-O-glucoside	$C_{21}H_{24}O_{11}$	500	$[M+H]^+$	453.1393	0.38	NA	2.36	2
23	Epicatechin-3'-O-glucoside	$C_{21}H_{24}O_{11}$	523	$[M+H]^+$	453.139	-0.35	NA	2.49	2
24	Epigallocatechin gallate	$C_{22}H_{18}O_{11}$	546	$[M+H]^+$	459.0918	-0.91	139.0396, 89.0606	2.62	1
25	Corniculatusin 3-glucoside	$C_{22}H_{22}O_{13}$	589	$[M+H]^+$	495.1133	-0.07	333.0608, 318.0373	2.86	2
26	Phosphatidylcholine lyso 18:2	$C_{26}H_{50}NO_7P$	1310	$[M+H]^+$	520.3402	0.74	184.0731, 86.0969	6.82	2
27	Procyanidin B1	$C_{30}H_{26}O_{12}$	495	$[M+H]^+$	579.1501	0.67	127.0393, 287.0553	2.33	1
28	Procyanidin B4	$C_{30}H_{26}O_{12}$	521	$[M+H]^+$	579.1499	0.34	127.0395, 287.0557	2.48	1
29	Sexangularetin 3-rhamnoside-7-glucoside	$C_{28}H_{32}O_{16}$	555	$[M+H]^+$	625.1764	0.13	317.0685	2.67	2
30	Kaempferol-malonyl-rhamnose-rhamnose	$C_{30}H_{32}O_{17}$	644	$[M+H]^+$	665.1707	-0.76	287.0556	3.17	2
31	Kaempferol 3-rutinoside-7-rhamnoside	$C_{33}H_{40}O_{19}$	511	$[M+H]^+$	741.2235	-0.2	287.0551	2.42	2
32	PC(C16:0/C18:3)	$C_{42}H_{78}NO_8P$	1611	[M+]	756.5537	-0.17	184.0738	7.74	2
33	Kaempferol-glucosyl-rhamnosyl-glucoside	$C_{33}H_{40}O_{20}$	504	$[M+H]^+$	757.2178	-0.98	287.0553	2.38	2
34	Procyanidin trimer 1	$C_{45}H_{38}O_{18}$	512	$[M+H]^+$	867.2134	0.33	NA	2.43	2
35	Procyanidin trimer 2	$C_{45}H_{38}O_{18}$	541	$[M+H]^+$	867.2135	0.47	NA	2.59	1
36	Soyasaponin Bc	$C_{47}H_{76}O_{17}$	1001	$[M+H]^+$	913.5145	-1.16		5.76	2

37	Soyasaponin γg	$C_{48}H_{74}O_{17}$	1139	$[M+H]^+$	923.4989	-1.06	423.3628, 141.0186	6.26	2
38	Dehydrosoyasaponin I	$C_{48}H_{76}O_{18}$	1070	$[M+H]^+$	941.5096	-0.85	421.3460, 85.0292	6.02	2
39	Soyasaponin βa	$C_{53}H_{82}O_{20}$	1076	$[M+H]^+$	1039.545	-2.1		6.04	2
40	Soyasaponin III	$C_{42}H_{68}O_{14}$	1006	$[M+H]^+$	797.4675	-0.88	423.3630, 141.0186	5.78	2
41	Naringenin glucuronide	$C_{21}H_{20}O_{11}$	715	$[M+H]^+$	449.1079	0.14	NA	3.57	2
42	Quercetin galactoside	$C_{21}H_{20}O_{12}$	596	$[M+H]^+$	465.1027	-0.05	303.0506	2.9	3
43	Quercetin diglucoside	$C_{27}H_{30}O_{17}$	495	$[M+H]^+$	627.1558	0.36	303.0503	2.33	3
44	PC(14:0/22:6)	$C_{44}H_{76}NO_8P$	1648	$[M+H]^+$	778.5378	-0.49	184.0730	7.95	2
45	PC(14:0/22:5)	$C_{44}H_{78}NO_8P$	1725	$[M+H]^+$	780.5537	-0.09	184.0735	8.21	2
46	LysoPE(0:0/18:2)	$C_{23}H_{44}NO_7P$	1311	$[M+H]^+$	478.2929	0.07	337.2734, 95.0860	6.81	2
47	LysoPE(0:0/16:0)	$C_{21}H_{44}NO_7P$	1370	$[M+H]^+$	454.2927	-0.17	313.2723, 95.0861	7.01	2
48	LysoPE(0:0/18:3)	$C_{23}H_{42}NO_7P$	1228	$[M+H]^+$	476.2771	-0.1	335.2576, 95.0859	6.53	2
49	LysoPC(18:1/0:0)	$C_{26}H_{52}NO_7P$	1426	$[M+H]^+$	522.3556	0.41	184.07321, 86.0970	7.2	2
50	LysoPC(16:0/0:0)	$C_{24}H_{50}NO_7P$	1376	$[M+H]^+$	496.3397	-0.11	184.07310, 86.0970	7.03	2
51	LysoPC(18:3/0:0)	$C_{26}H_{48}NO_7P$	1376	$[M+H]^+$	518.3218	-4.51	184.07326, 86.0970	7.03	2
52	LysoPC(20:5/0:0)	$C_{28}H_{48}NO_7P$	1317	$[M+H]^+$	542.3216	-4.6	337.27280, 86.0970	6.83	2
53	MG(0:0/20:4)	$C_{23}H_{38}O_4$	1713	$[M+H]^+$	379.2842	-0.16	347.2584, 177.1275	8.17	2
54	Kaempferol 3-O-arabinoside	$C_{20}H_{18}O_{10}$	607	$[M+H]^+$	419.0972	-0.14	287.0554	2.96	3
55	MG(0:0/18:3)	$C_{21}H_{36}O_4$	1361	$[M+H]^+$	353.2685	-0.42	297.0387, 95.0859	6.98	2
56	Kaempferol 3-sophoroside 7-glucoside	$C_{33}H_{40}O_{21}$	497	$[M+H]^+$	773.2127	-1.01	287.0388	2.34	2

57	Tragopogonsaponin G	$C_{56}H_{82}O_{21}$	1089	$[M+H]^+$	1091.539	-2.65	331.1007, 445.1321	6.06	2
58	Epigallocatechin	$C_{15}H_{14}O_7$	500	$[M+H]^+$	307.0813	0.45	215.1393, 243.1706	2.35	1
59	Procyanidin trimer 3	$C_{45}H_{38}O_{18}$	463	$[M+H]^+$	867.2136	0.59	NA	2.15	3
60	Prodelphinidin trimer	$C_{45}H_{38}O_{20}$	482	$[M+H]^+$	899.2028	-0.11	NA	2.26	2

rt: retention time

***m/z*:** mass-to-charge ratio

Identification confidence[71]**:** 1 = matching standard, 2 = high MS/MS spectra library match, and 3 = MS1 *m/z* database match.
